# Natural Toolbox–Chemical Engineering Aspect and High‐Value Applications of Janus Cellulose Nanomaterials

**DOI:** 10.1002/advs.202500820

**Published:** 2025-05-21

**Authors:** Yu Zhao, Shengjie Wang, Zongchen Wei, Shiming Qiu, Guofu Zhou, Jr‐Hau He, Xuezhu Xu

**Affiliations:** ^1^ Guangdong Provincial Key Laboratory of Optical Information Materials and Technology and Institute of Electronic Paper Displays South China Academy of Advanced Optoelectronics South China Normal University Guangzhou 510006 China; ^2^ Chongzuo Key Laboratory of Comprehensive Utilization Technology of Manganese Resources Guangxi Key Laboratory for High‐value Utilization of Manganese Resources College of Chemistry and Biological Engineering Guangxi Minzu Normal University Chongzuo Guangxi 532200 China; ^3^ Chongzuo Key Laboratory of High‐value Utilization of Sugarcane Resources Chongzuo Guangxi 532200 China; ^4^ COFCO Corporation Chongzuo Sugar Co., Ltd Chongzuo Guangxi 532200 China; ^5^ National Center for International Research on Green Optoelectronics South China Normal University Guangzhou 510006 China; ^6^ Department of Materials Science and Engineering City University of Hong Kong, Kowloon Tong Hong Kong SAR China

**Keywords:** amphiphilicity, cellulose nanomaterials, hydrogen bonding, hydrophobic interaction, Janus

## Abstract

Nanotechnology has emerged as a transformative force, enabling the manipulation and engineering of materials at the nanoscale level, which has led to the discovery and development of novel materials with unique properties and functionalities. Janus cellulose nanomaterials, a product of nanotechnology, have attracted significant attention. This article aims to address the current lack of fundamental mechanistic understanding in Janus cellulose by investigating the intrinsic relationship between structural design and functional performance. Specifically, it begins by elucidating the construction principles of Janus cellulose nanomaterials, with a particular focus on how their asymmetric architectures impart anisotropic physicochemical properties, such as interfacial tension modulation, directional interactions, and selective transport. By integrating multiscale modeling approaches—including molecular dynamics simulations and density functional theory calculations—the underlying interfacial behaviors and assembly pathways are revealed, providing theoretical insight into their conformational stability and dynamic response mechanisms. It also explores how surface functionalization and selective chemical modification strategies can be leveraged to finetune hydrophilicity/hydrophobicity balance and interfacial activity, thereby enabling precise control over Janus cellulose interface configuration and functional attributes. On this basis, it further examines non‐covalent driving forces—including electrostatic interactions, van der Waals forces, and hydrogen bonding—within the self‐assembly process, and systematically maps the relationship between assembly conditions and structural evolution. This work establishes a comprehensive structure–driving force–assembly process–property framework, offering theoretical support and design guidance for the development of high‐performance Janus cellulose nanomaterials in advanced applications such as flexible electronics, smart sensing systems, controlled drug delivery, and energy conversion and storage.

## Graphical abstract

The *Tai Ji* symbol from China represents the co‐existence of two opposite substances, similar to quantum entanglement. The term *Janus* comes from ancient Roman religion and mythology. Janus, the god of beginnings, transitions, etc., has two faces, symbolizing duality. *Janus* cellulose nanomaterials show structural duality. Their crystal planes have different properties, leading to amphiphilicity due to the presence of hydroxyl and ‐CH groups. Structural duality, amphiphilicity, chemical modification, self–assembly, industrial scalability, mechanisms, and simulation‐based understanding of these materials are closely related. They determine the use of materials as a “natural toolbox” source and their potential in chemical engineering and high–value applications. Chemical modification can enhance amphiphilicity, self–assembly is key for functional materials, and understanding mechanisms and using simulations help optimize the materials for applications like pressure sensing and electromagnetic shielding.

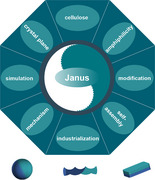

## 1 Introduction

### 1.1 Cellulose Nanomaterials

**Figure 1:**
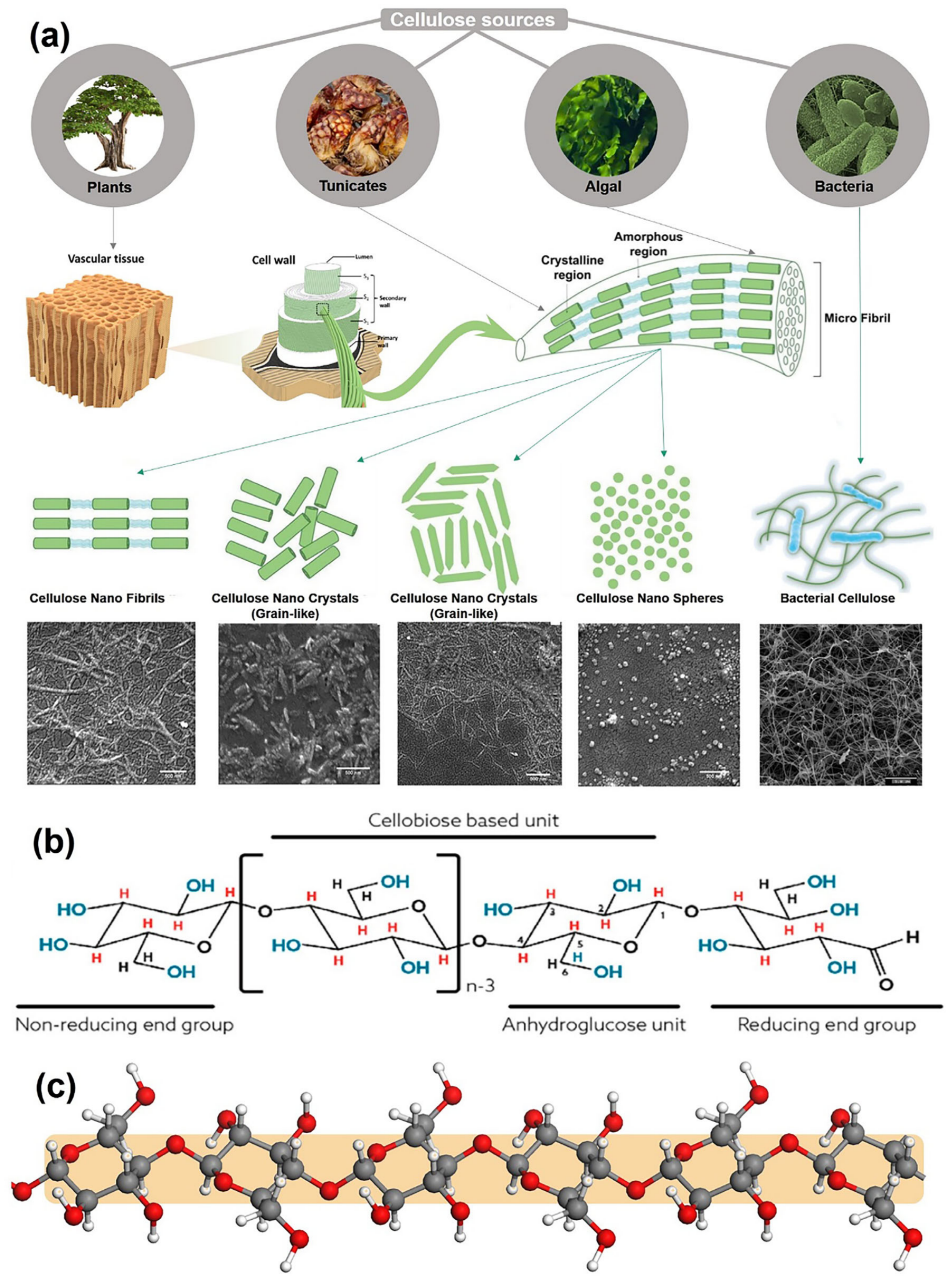
a) Schematic representation of the cellulose source, plant cell wall, and cellulose fiber structure. CNFs, CNSs, and CNCs are extracted from cellulose fibers using mechanical process, cellulase, and chemical methods (oxidation or acid hydrolysis), respectively.^[^ ^13^, ^14^ ^]^ b) Chemical structure diagram of cellulose, and reducing end group in the open‐chain aldehyde form (right end) is in equilibrium with a hemiacetal ring form.^[^ ^15^ ^]^ c) Modeling structure of cellulose molecule. The red ball represents the O, the black ball represents the C, and the white ball represents the H. Reprinted with permission from ref.[13] Copyright 2023: ASC; Reprinted with permission from ref.[14] Copyright 2024: Springer; Reprinted with permission from ref.[15] Copyright 2021: CUP.

The term “cellulose,” first coined by Payen at the French Academy of Sciences in 1839 to describe the structural polysaccharide in plant cell walls, has evolved into a cornerstone of nanotechnology.^[^ ^1^ ^]^ As illustrated in **Figure** **1**a, cellulose is sourced not only from plants but also from tunicates, bacteria, and algae, each offering unique nanostructural features. Today, cellulose nanomaterials (CNMs) encompass six primary forms: cellulose nanocrystals (CNCs), nanofibrils (CNFs), tunicate CNCs (t‐CNCs), bacterial cellulose (BC), algal cellulose (AC),^[^ ^2^, ^3^, ^4^, ^5^, ^6^, ^7^, ^8^ ^]^ and specialized derivatives like nanocellulose spheres (NCSs) or sheets.^[^ ^9^ ^]^ These can also be often called nanocellulose by many researchers since the year 2000.

In fact, cellulose nanomaterials, abbreviated into CNMs, and nanocellulose are closely related terms often used in materials science, but they carry distinct nuances. Nanocellulose serves as an umbrella term for cellulose‐derived structures engineered at the nanoscale (1–100 nm), including three primary subtypes: cellulose nanocrystals (CNCs), rod‐like particles produced through acid hydrolysis that retain crystalline regions of cellulose; cellulose nanofibrils (CNFs), flexible fibrils generated via mechanical processing (e.g., grinding) combined with chemical or enzymatic pretreatment; and bacterial cellulose (BC), a highly pure, crystalline 3D network biosynthesized by bacteria. In contrast, cellulose nanomaterials represent a broader category encompassing not only nanocellulose but also hybrid or functionalized materials (e.g., composites, coatings, or nanoyarns) that integrate cellulose nanostructures with other components like polymers or nanoparticles.

In Figure 1b, cellulose molecules can undergo configurational tautomerism between aldehydes and hemiacetals at the reducing end.^[^ ^10^ ^]^ Chemically modifying the reducing terminal is a promising avenue for cellulose materials research. From the perspective of the cellulose molecular structure, the side‐chain hydroxyl groups provide abundant modification sites, while the in‐chain hydrogen bonds between neighboring ring molecules form a stable linear polymer chain.^[^ ^11^ ^]^ In Figure 1c, we show the molecular structure of cellulose sketched with Materials Studio, with the model at its minimum energy state. The exposed hydroxyl groups around the cellulose backbone give it hydrophilic properties, while the carbon backbone and ─CH groups contribute to its hydrophobic nature. These materials form a versatile “natural toolbox,” enabling nanostructures from 0D (e.g., CNCs) to 3D (e.g., BC networks),^[^ ^12^ ^]^ with transformative potential for advanced applications, including Janus cellulose nanomaterials—asymmetric systems with dual functionalities.

### 1.2 Janus Cellulose Nanomaterials

Pierre‐Gilles de Gennes proposed the concept of “Janus particles” as anisotropic nanomaterials to the scientific community in his Nobel Prize‐winning lecture “Soft Matter.”^[^ ^16^, ^17^ ^]^ They are defined as anisotropic structures with two or more physical properties, chemical, morphology, magnetism, and optical properties.^[^ ^18^, ^19^, ^20^, ^21^ ^]^ Since the introduction of the Janus structure concept, researchers have leveraged its distinctive characteristics to address scientific challenges across various fields, including fundamental physics, chemistry, biomedical sciences, and materials science. Initially, Janus structures were predominantly applied in nanotechnology, interface science, catalysis, and biomedicine. However, with ongoing research, their applications have progressively extended to high‐tech areas such as flexible electronics, sensors, and optoelectronic devices.^[^ ^22^, ^23^, ^24^, ^25^, ^26^, ^27^ ^]^ Research on cellulose nanomaterials primarily focuses on the enhancement, functional integration, and green processing capabilities derived from their uniform structure. In contrast, Janus cellulose nanomaterials, by incorporating interface asymmetry, introduce directional interface control and multifunctional response mechanisms, showcasing distinct advantages in high‐end smart materials, flexible electronics, and energy management. This shift represents a critical transformation in cellulose materials research, transitioning from symmetric structures to functional differentiation, marking a key advancement in the development of advanced functional materials. The Janus concept of cellulose nanomaterials was first cited in a 2013 paper by Arcot et al.,^[^ ^28^ ^]^ showing the functionalization of CNCs reduced‐end mercaptan with the specific adsorption of silver nanoparticles to form Janus‐like colloidal rod‐globule polymers.

**Figure 2:**
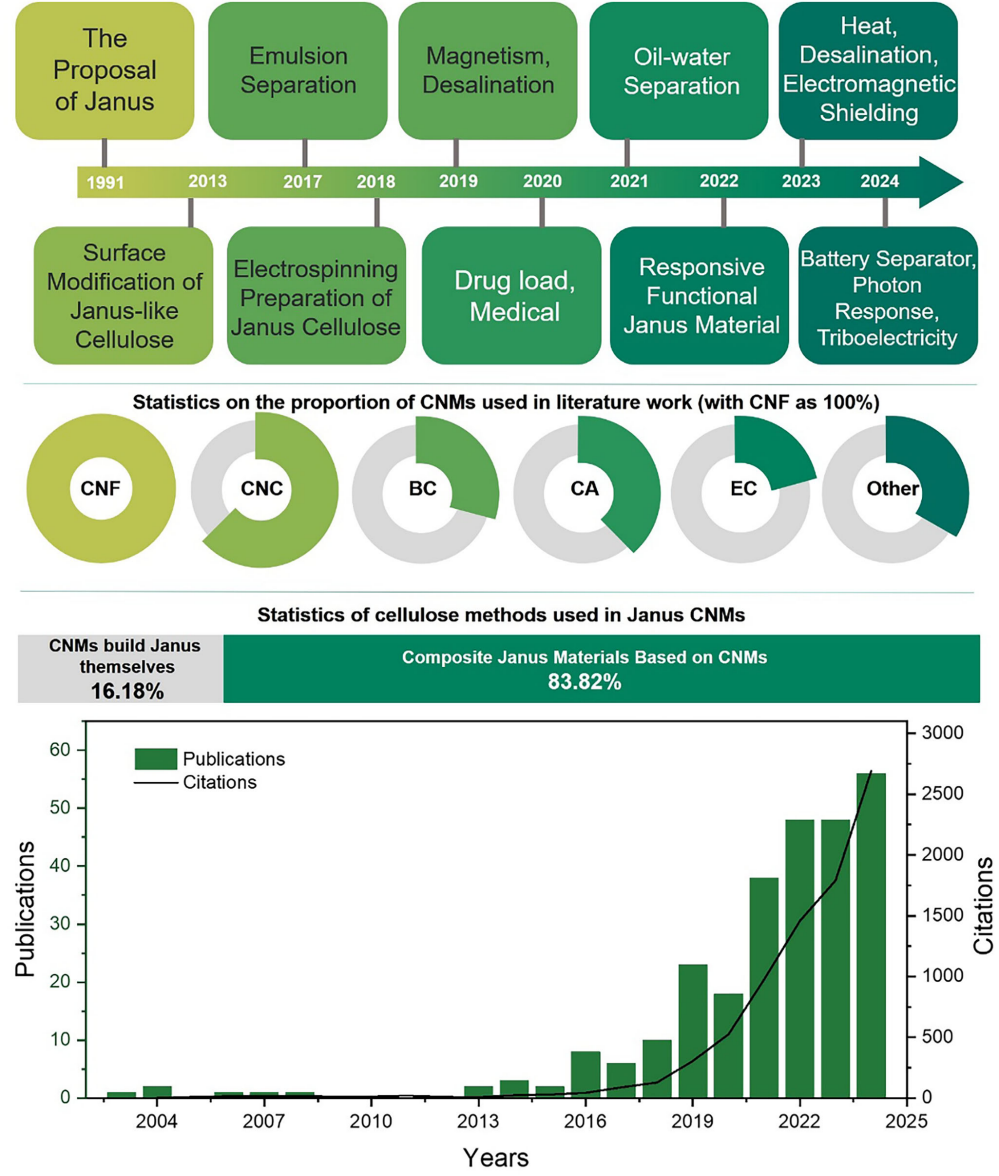
Applications, cellulose types, preparation methods schematic, and publication trends (based on keywords “cellulose” and “Janus”) of Janus cellulose nanomaterials.^[^ ^20^, ^28^, ^29^, ^30^, ^31^, ^32^, ^33^, ^34^, ^35^, ^36^, ^37^, ^38^, ^39^, ^40^, ^41^, ^42^, ^43^, ^44^, ^45^, ^46^, ^47^, ^48^, ^49^, ^50^, ^51^, ^52^, ^53^, ^54^, ^55^, ^56^, ^57^, ^58^, ^59^, ^60^, ^61^, ^62^, ^63^, ^64^, ^65^, ^66^, ^67^, ^68^, ^69^, ^70^, ^71^, ^72^, ^73^, ^74^, ^75^, ^76^, ^77^, ^78^, ^79^, ^80^, ^81^, ^82^, ^83^, ^84^, ^85^, ^86^, ^87^, ^88^, ^89^, ^90^, ^91^ ^]^ (CA: Cellulose acetate, EC: ethyl cellulose).

**Figure** **2** summarizes recent research trends in Janus cellulose nanomaterials. There are presently two trends in utilizing CNMs to construct Janus materials. One involves leveraging the facile modification and functionalization properties of CNMs to create Janus materials.^[^ ^28^, ^29^, ^30^ ^]^ Recently, Kondo's team developed Janus‐type cellulose surfaces from chemically unmodified CNFs using aqueous counter collision.^[^ ^31^, ^32^, ^33^ ^]^ This highlights the potential of the inherent Janus properties of CNMs for constructing large‐scale, uniform materials. An alternative approach involves synthesizing Janus composite materials through traditional methods, utilizing cellulose as the substrate, and introducing various materials.^[^ ^34^, ^35^, ^36^ ^]^ We will offer a comprehensive and comparative analysis of specific synthesis methods later. While the latter strategy prevails in the majority of Janus cellulose material research (constituting 83.82%), it may not be the most effective. Constructing Janus characteristic materials by leveraging the inherent advantages of cellulose itself is a more appealing approach.

In natural cellulose crystals, flakes are stacked by van der Waals forces resulting from hydrogen bonds between planes, with hydrogen bond energies ranging from 17 to 30 kJ mol^−1^ and intermolecular hydrogen bond energies ≈20 kJ mol^−1^.^[^ ^13^ ^]^ The surface roughness (5 nm) of the film formed by CNMs is superior to that of polymer plastics, and its maximum loading stress (200–400 MPa) far exceeds that of paper (6 MPa). CNMs exhibit a good coefficient of thermal expansion (≈0.1 ppm·K⁻¹) and excellent thermal stability within the range of 260–370 °C, with a strength comparable to that of low‐alloy materials (20–50 GPa).^[^ ^92^, ^93^ ^]^ CNMs exhibit excellent biocompatibility, targeted loading potential, a large surface area, outstanding mechanical properties, and good wettability, enabling them to carry high‐quality loads (9.2 mg cm^−^^2^). Sriplai et al.^[^ ^94^ ^]^ employed a 3D nanofiber network of BC fibers to diffuse and anchor water‐based Fe₃O₄ nanoparticles in a ferromagnetic fluid solution, successfully preparing layered magnetic BC nanocomposites.

Currently, reviews on Janus cellulose primarily focus on applications such as emulsion separation, medical uses, thermal management, preparation technologies, colloidal properties, and asymmetric structures.^[^ ^39^, ^95^, ^96^ ^]^ However, discussions on its fundamental mechanisms in fields like optoelectronics, chemistry, and fine control remain limited. This lack of detailed exploration hinders the provision of clear guidance for researchers in the construction of Janus cellulose nanomaterials and their theoretical foundations in high value‐added applications, such as pressure sensing, electromagnetic shielding, optoelectronic devices, and efficient energy management.

In this study, we investigate the chemical engineering aspects and high‐value applications of the Janus face of cellulose nanomaterials, focusing on their emerging characteristics and applications. While previous reviews have touched on related topics, our goal is to provide more in‐depth evidence and value‐added applications aligned with our institute's expertise in physics. We analyze existing literature on the asymmetric modification of cellulose nanomaterials (CNMs) and relate their amphiphilicity to JANUS features in various domains. We summarize molecular‐level driving forces that explain Janus properties, such as hydrogen bonding and hydrophobic interactions. By integrating molecular simulation data, we substantiate the Janus characteristics and emphasize chemical modifications that enhance performance and provide valuable tools for advancing cellulose nanomaterials in high‐value applications.

## 2 Intrinsic Janus Characteristics of CNMs

### 2.1 Discovery and Study of the Amphiphilic Properties of Cellulose

Cellulose has traditionally been considered hydrophilic due to its abundant hydroxyl (‐OH) groups, which confer strong affinity for water. However, advancements in nanotechnology, particularly with cellulose nanomaterials, have transformed this understanding. At the nanoscale, cellulose can exhibit both hydrophilic and hydrophobic properties, and through surface modification and structural engineering, its amphiphilicity—coexistence of hydrophilic and hydrophobic characteristics—can be significantly enhanced. In general, the total surface energy of cellulose is between 50 and 60 mN m^−1^ although the contribution of the dispersive and polar components can be significantly different, depending on the type of cellulose in question.^[^ ^13^ ^]^ An intriguing example supporting the amphiphilicity of cellulose glucose rings is cyclodextrin. Cyclodextrin exhibits high water solubility and has the ability to internally bind very non‐polar molecules.^[^ ^97^ ^]^ Bruel et al. conducted research on the amphiphilicity of cellulose. According to Hansen solubility parameters (HSP measured in 59 solvents and binary mixtures: δ_D_; δ_P_; δ_H_), the relationship between solubility data and the exposed crystalline surface of wood‐based H_2_SO_4_ hydrolyzed CNCs was proposed. Two sets of cohesive force parameters correspond to polar surfaces (18.1; 20.4; 15.3) ± (0.5; 0.5; 0.4) MPa^1/2^ and mild non‐polar surfaces (17.4; 4.8; 6.5) ± (0.3; 0.5; 0.6) MPa^1/2^, aligning with the cellulose Iβ exposure of (110) and (110) surfaces of nanocrystals, as well as (200) surfaces.^[^ ^98^ ^]^ But in fact, cellulose can be well used in the stability of emulsion.^[^ ^99^, ^100^, ^101^ ^]^ Thus, if the macroscopic properties are extrapolated to isotropic nanoparticles, our observations appear to be surprising. Understanding such behavior necessitates evaluating the extent of edge truncation exposure in crystals, where β‐linked anhydroglucoses lie flat, exposing the axial hydrogen, while all hydroxyls remain equatorial. Structural studies have previously reported a partial hydrophobic character of cellulose.^[^ ^102^ ^]^

### 2.2 Experimental and Theoretical Evidence of Janus Properties in CNMs at the Micro Scale

**Figure 3:**
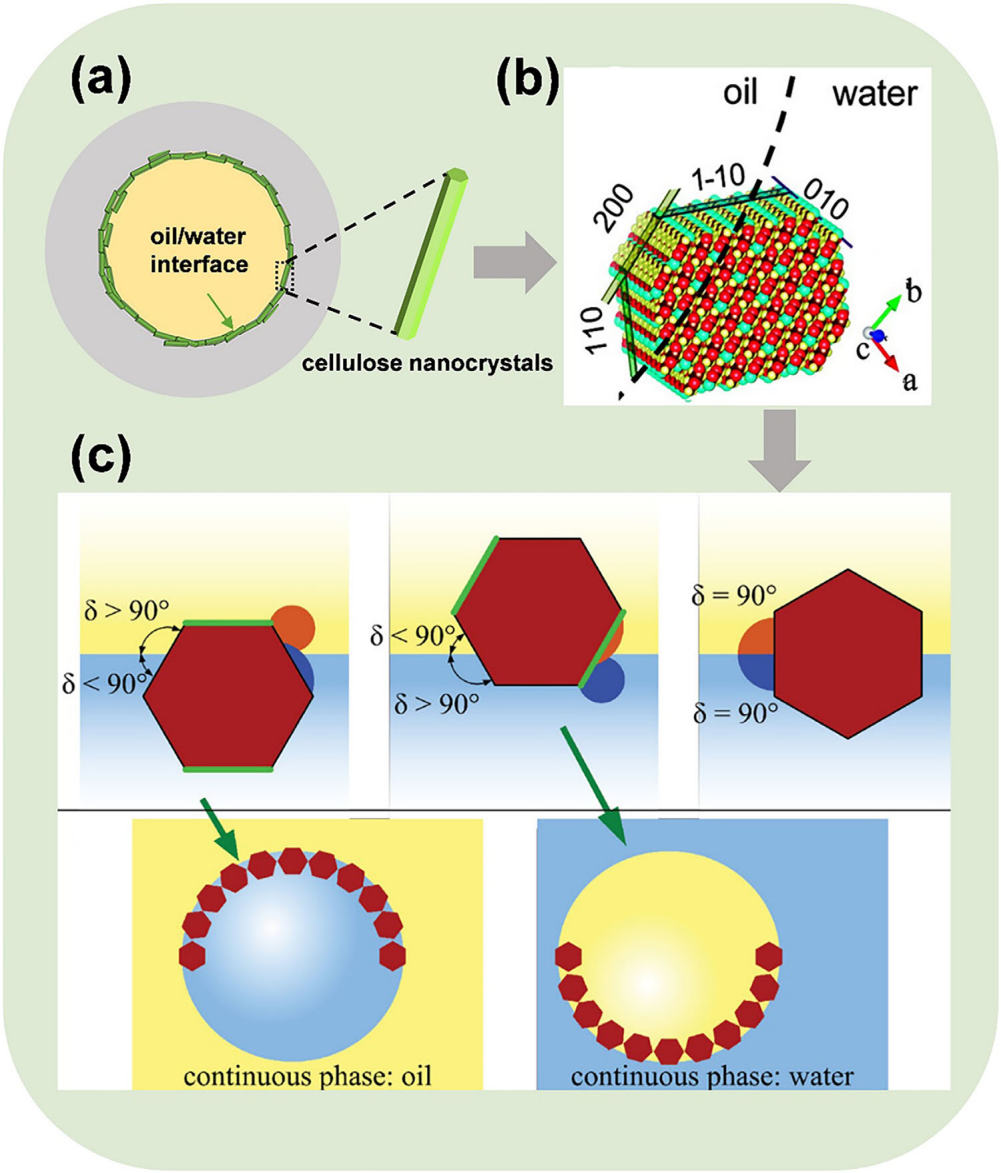
a) Diagram illustrating the stabilization of emulsion by cellulose nanocrystals. b) Diagram depicting the mechanism of oil‐water interface stabilization facilitated by an amphiphilic plane. c) Formation of micelles.^[^ ^109^, ^112^ ^]^ Reprinted with permission from ref.[109] Copyright 2012: ACS; Reprinted with permission from ref.[112] Copyright: 2021, Wiley.

In the initial phase, unmodified cellulose was employed to assess various factors influencing lotion stability, with a significant emphasis on its amphiphilic properties.^[^ ^103^ ^]^ CNC has exhibited Janus‐like behavior, although some attribute it to steric effects. Cellulose nanocrystals can stabilize Pickering systems through their Janus properties, forming a protective layer at the oil‐water interface. Simultaneously, they create an associated structure in the continuous phase, enhancing their viscoelastic properties and preventing flocculation (**Figure** **3**a).^[^ ^95^, ^101^, ^104^ ^]^ Kalashnikova et al. prepared three O/W Pickering emulsions stabilized by the CNCs from cotton (CNC, aspect ratio of 13), bacterial cellulose (BC, aspect ratio of 47) and Cladophora (AC, aspect ratio of 160), respectively.^[^ ^105^ ^]^ BC is known to be organized in an Iα rich crystal lattice and composed of ribbon‐like microfibrils.^[^ ^106^ ^]^ Consequently, different proportions of crystal planes are exposed. Unmodified quasi‐neutral bacterial cellulose nanocrystals (BCN) effectively stabilize the oil‐water interface.^[^ ^107^ ^]^ However, when sulfated cotton cellulose nanocrystals (CCN) were used, no emulsion was observed. The sulfate charge of cotton nanocrystals hydrolyzed by sulfuric acid ranges from 0.155 to 0.41 e mm^−2^.^[^ ^108^ ^]^ Investigating the contribution of electrostatic repulsion to the amphiphilic plane. The Capron team treated cellulose sulfate with hydrochloric acid, resulting in a sequential decrease in surface charge density. Attaining a plateau value of 0.017 ± 0.002 e mm^−2^.^[^ ^109^ ^]^ The hydrolysis with HCl does not induce significant changes in the overall surface area or crystal structure of the nanocrystals, except for variations in charge density. Cellulose crystals may exhibit an orientation at the interface, leading to some planes with sulfate groups and others without charges, influenced by the interaction of hydrophobic sites exposed on the surface.^[^ ^32^ ^]^

In fact, several studies have been based on the interactions with hydrophobic sites exposed on cellulosic surfaces, for example, the modeling study of aromatic compounds, direct dyes used as molecular probes to characterize cellulose substrates, or dyes such as calcofluor that may intercalate within the lattice.^[^ ^110^, ^111^ ^]^ An even better example is the identification of genetically conserved aromatic amino acids involved in some fungal carbohydrate‐binding modules that may stack at their flat exposed surface onto “hydrophobic” surfaces.^[^ ^102^ ^]^ These experiments tend to demonstrate that some parts of the nanocrystals expose less polar groups at the surface. This character has been attributed to one crystalline plane for which axial CH moieties are directly exposed at an edge truncation at the surface of the nanocrystals. However, some equivalent planes exist between Iα and Iβ allomorphs that are similar in terms of hydrophilic affinity, roughness, and surface energy. The various surfaces can therefore be divided into three families (Figure 3b).

According to the amount of ‐OH and hydrophobic ‐CH exposed in different crystal planes, the hydrophilic and hydrophobic ratios of cellulose nanocrystals can be regulated by modification. The particles formed have different affinity for the oil phase and the water phase, which can regulate the emulsion type (oil‐in‐water O/W and oil‐in‐water W/O), as shown in Figure 3c.

CNC particles with high hydrophobicity will penetrate deep into the oil phase to minimize excess surface energy. Janus CNC particles, which are more hydrophilic, also have the opposite effect. However, if hydrophilic and hydrophobic levels are symmetrically distributed, the observations of the two hydrophilic planes in opposite directions in the aqueous and oil phases appear to be contradictory.^[^ ^95^, ^112^ ^]^ The stability and functionality of the emulsion can be effectively enhanced by modification and coating of cellulose nanomaterials.

**Figure 4:**
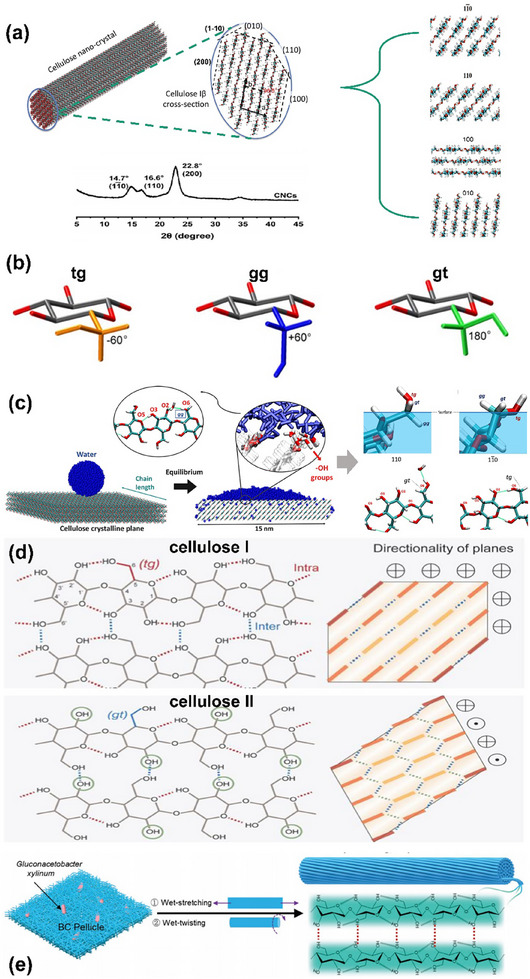
a) schematic of the (010), (110), (100), (200), (110), and (1‐10) crystal faces in plant nanocrystals Iβ generated by the “Cellulose Builder” toolkit. Simultaneously showcasing the molecular arrangement details of different crystal planes. Cellulose nanocrystals Iβ (200), (110), and (1‐10) XRD peaks.^[^ ^115^, ^130^ ^]^ b) The conformational types resulting from the rotation of hydroxyl groups in cellulose molecules.^[^ ^119^ ^]^ c) The impact of molecular surface conformation on wetting and its elucidation.^[^ ^115^ ^]^ d) The mutual conversion of cellulose Iβ to cellulose II involves the regulation of molecular structure and conformation through hydrogen bonding.^[^ ^131^ ^]^ e) Ordered stacking of cellulose molecules.^[^ ^132^ ^]^ Reprinted with permission from ref.[115] Copyright: 2021, ACS; Reprinted with permission from ref.[119] Copyright: 2006, Elsevier; Reprinted with permission from ref.[130] Copyright: 2019, Elsevier; Reprinted with permission from ref.[131] Copyright: 2021, Springer; Reprinted with permission from ref.[132] Copyright: 2017, Wiley.

The monoclinic polymorph (Iβ) has three major lattice planes (200), (110), and (1−10), which is confirmed by the presence of three large peaks in the X‐ray diffraction spectrum (**Figure** **4**a).^[^ ^113^, ^114^ ^]^ In addition to comprehending the Janus characteristics of cellulose through the mentioned experimental perspective, the current research trend involves modeling CNMs by regulating molecular conformation and arrangement through simulation techniques.^[^ ^112^, ^115^ ^]^ This approach allows for the study of crystal planes, surface energy, wettability, and other related issues.^[^ ^116^, ^117^, ^118^, ^119^, ^120^ ^]^ The contact angle (CA) serves as a visual representation of the wetting differences among various crystal faces of cellulose nanocrystals with Janus properties. This angle can be experimentally measured or calculated using a formula.^[^ ^121^, ^122^ ^]^ Given that cellulose materials are predominantly solid, this study focuses on solid‐liquid contact theory, analyzing the surface of a specific area. Considering the changes in system energy, the degree of wetting should be measured by the adhesion work, where W_a_ is equal to the change in surface Gibbs free energy during this process: W_a_ = Δ G^σ^ = (γ_sl_ − γ_lv_ − γ_sv_). The greater the adhesion work, the stronger the liquid‐solid bond, indicating increased wettability. Simultaneously, the Young's equation is employed to measure the CA (θ) through surface tension, providing an evaluation of the degree of wetting: cos θ = (γ_sv_ − γ_sl_)/γ_lv_. γ_lv_, γ_sv_, γ_sl_ respectively represent the interfacial tension of liquid‐gas, solid‐gas, and solid‐liquid interfaces.^[^ ^121^ ^]^

The study provides valuable insights into the amphiphilic properties and Janus behavior of CNCs, highlighting their ability to stabilize Pickering emulsions through surface modification. The use of different cellulose sources, such as bacterial and cotton cellulose, demonstrates the impact of crystal structure and surface charge on emulsion stability. A notable strength of this research is its integration of molecular simulation techniques to explore the crystal planes and wettability of CNCs. However, the study could further investigate the influence of modification types on long‐term emulsion stability and expand the scope of practical applications. Future work could focus on optimizing CNC surface modification for enhanced emulsion performance and developing scalable techniques for industrial applications.

### 2.3 Regulating the Microscopic Janus Structure of CNMs: Driving Forces and Mechanisms

#### 2.3.1 Hydrogen Bonding

Hydrogen bonding (HB) is a key factor influencing the physical and chemical properties of cellulose nanocrystals, particularly in aqueous environments, where it governs intermolecular interactions. The hydrogen bonds between water molecules and the hydroxyl groups of cellulose play a crucial role in determining its conformation.^[^ ^123^ ^]^ In the sheet‐like structure of cellulose, the anisotropic molecular arrangement aligns hydroxyl groups parallel to the (200) lattice plane, contributing to the formation of hydrogen bond networks. This results in wetting properties similar to those of amorphous cellulose. However, the crystalline structure, particularly the Iβ isomer, imparts hydrophobic characteristics to cellulose. In natural cellulose crystals, hydrogen bonds stack the sheet‐like structures, further influencing cellulose's physical and chemical properties through van der Waals interactions between the planes.^[^ ^124^ ^]^

Molecular dynamic (MD) simulations of Type I nanocrystalline cellulose reveal its amphiphilic nature (Figure 4a). The hydrophilic (110) face, predominant in native fibers, has a contact angle of 43°, while the hydrophobic (100) face has a contact angle of 95°.^[^ ^120^ ^]^ The (200) face, containing C─H groups, is hydrophobic, whereas the (110) and (1−10) faces, rich in hydroxyl groups, are hydrophilic.^[^ ^95^, ^115^, ^120^ ^]^ Yurtsever unveiled the molecular details of the arrangement of cellulose chains on the surface of individual CNCs in water using atomic force microscopy in conjunction with MD simulations.^[^ ^125^ ^]^ The distribution of water‐oxygen density reveals notable differences in the molecular details of water structures at various interfaces, reflecting the heterogeneity of interactions between CNCs and water at the molecular level.

Amphiphilicity exists at both the molecular and supramolecular levels, guided by structure and conformational order. This allows for conformational changes to adapt to the surrounding medium.^[^ ^126^ ^]^ The three most probable rotational positions of the hydroxymethyl group are defined by ascertaining the placement of the O6‐C6 bond with respect to the O5‐C5 and C4‐C5 bonds: if O6‐C6 is gauche to O5‐C5 and trans to C4‐C5, then the conformation is called **gt**, while the other two conformations are referred to as **gg** and **tg** (Figure 4b).^[^ ^127^ ^]^ When the cellulose surface is fully hydrated, the gt conformation that arises seems to be associated with an increased wetting of the Iβ (110) cellulose plane. On an Iβ (1‐10) surface, both **tg** and **gt** conformations are not highly exposed to water molecules. Therefore, the less hydrophilic surfaces exhibit a higher population of hydroxymethyl conformations that are less prone to interact with water. The same reasoning can also be used to explain the difference in gg populations between these surfaces. Hydroxymethyl groups in gg conformations display significant water accessibility on an Iβ (1‐10) surface. On the contrary, they are deeply buried in the Iβ (110) surface and thus are not positioned to form cellulose−water nor cellulose−cellulose hydrogen bonds (Figure 4c). This demonstrates the influence of the hydroxymethyl group conformation on the crystal surface wettability. In natural cellulose crystals, molecular chains are interconnected through planar van der Waals forces and hydrogen bonds. Within the sheet, the intramolecular hydrogen bonds in the native cellulose I crystal are between HO(C3) − HO(C5) and HO(C2) − HO(C6), whereas the major intermolecular bond forms between HO(C3) and HO(C6) (Figure 4d). Overall, the hydrogen bond energy of cellulose ranges from 17 to 30 kJ mol^−1^, and the intermolecular hydrogen bond energy is approximated to be ≈ 20 kJ mol^−1^.^[^ ^117^ ^]^ The crystal structure of cellulose is controlled by conformations such as gg, gt, tg, etc.^[^ ^13^ ^]^ Previous work has shown that alkali treatment can transform cellulose I into cellulose II.^[^ ^128^ ^]^ In other words, cellulose nanocrystals have the potential for directional regulation. If we further reveal their stacking patterns and even regulate their growth direction from a molecular perspective, we may design more controllable and intelligent cellulose materials (Figure 4e).

Molecular dynamics simulations reveal that cellulose I nanocrystals exhibit amphiphilicity, with the (110) face showing a hydrophilic contact angle of 43° and the (100) face hydrophobic at 95°. Hydroxymethyl group conformations (gg, gt, tg) significantly influence surface wettability. The gg conformation enhances water accessibility on the (1‐10) face, while gt dominates hydration on the (110) face. Hydrogen bonding, with energies ranging from 17–30 kJ mol^−1^, underpins the structural stability. These insights highlight the molecular‐level tunability of cellulose surfaces for advanced material design.

And due to the hydrogen bonding between the hydroxyl groups in the equatorial direction (axial), it becomes important to study the exposed hydroxyl groups on the surface. Using the nanocrystal dimensions of 131 ± 10 × 13 ± 1 nm reported by Abitbol et al., it can be calculated that the number of hydroxyl groups on the surface of the cellulose nanocrystals varies between 1.57 mmol g^−1^ (0.51 mmol g^−1^ primary) and 1.82 mmol g^−1^ (0.59 mmol g^−1^ primary) for this dataset.^[^ ^129^ ^]^ In our previous research, we explored the modification of cellulose and its derivatives, leading to the concept of modifying CNMs materials to construct Janus materials.^[^ ^9^ ^]^ We found that most of the side group modifications do not affect the crystal form of cellulose nanocrystals, while most of the current Janus cellulose side group modifications are macroscopic, and Janus materials are not constructed from the nanostack level. Therefore, the focus of our review here is still on the reduction end. Of course, we expect that with the development of nanotechnology, we can carry out controllable Janus modification of CNMs molecular side chains at a more microscopic scale (rather than macroscopic), which will make an important contribution to promoting the development of nanotechnology.

#### 2.3.2 Hydrophobic Interaction

The layered stacking of cellulose nanocrystals is driven not only by hydrogen bonds but also by hydrophobic interactions, which arise from the release of structural water molecules.^[^ ^123^ ^]^ To understand the molecular mechanism of cellulose self‐assembly, it is essential to recognize that natural cellulose exhibits a high degree of polymerization (DP), resulting in a limited entropy gain during dissolution and consequently lower solubility. The structural rigidity of cellulose, combined with the presence of its hydrophobic regions, restricts entropy increase between molecules, preventing the generation of negative free energy changes upon dissolution in water. From a thermodynamic perspective, this suggests that cellulose dissolution in water is thermodynamically unfavorable.^[^ ^133^ ^]^

An interesting study performed by Bergenstråhle‐Wohlert et al.^[^ ^134^ ^]^ combines MD simulations and solid‐state NMR on cellulose in water and in aqueous urea solutions. The authors found that the local concentration of urea is significantly enhanced at the cellulose/solution interface. Radial distribution functions reveal that urea molecules have a preferential orientation, with its “hydrophobic part”, the nitrogen atoms, pointing in the direction of the cellulose backbone, and its hydrophilic part, the carbonyl group, pointing away from it.

**Figure 5:**
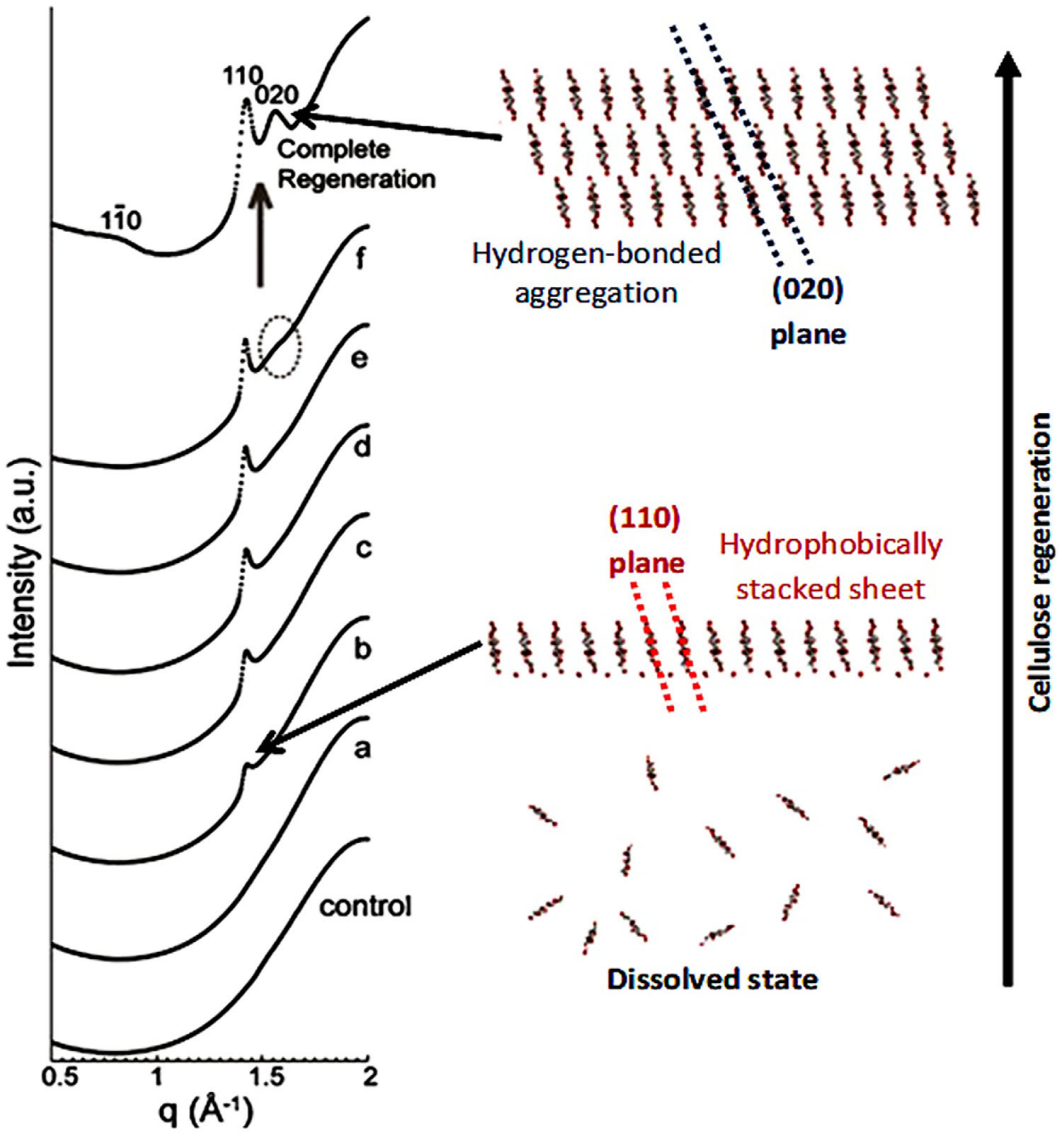
Synchrotron X‐ray diffraction profiles of cellulose solution under regeneration by 5 wt.% aq. Na_2_SO_4_, control: 10 wt.% cellulose solution without coagulant; a) measured at 2 mm away from the boundary with coagulant and 180 min after coagulant introduction; b) 2.5 mm, 195 min; c) 1.75 mm, 190 min; d) 1.5 mm, 185 min; e) 1.5 mm, 200 min; f) 1.5 mm, 270 min, and complete regeneration: 1.5 mm, 1 week. q denotes the scattering vector (2π/d).^[^ ^137^ ^]^ Reprinted with permission from ref.[137] Copyright: 2012, Elsevier.

In perfect agreement with this, Xiong et al.,^[^ ^135^ ^]^ while working in the same NaOH/urea system, clearly state that the addition of urea in the NaOH solvent can reduce the hydrophobic effect of cellulose since urea may play its role through interacting with the hydrophobic part of cellulose. Lina Zhang's^[^ ^136^ ^]^ group has been instrumental in investigating the effect of urea on cellulose solubility. An interesting study has been presented by Isobe et al.^[^ ^137^ ^]^ on the regeneration of cellulose, either using a coagulant or upon heating, in an aqueous alkali‐urea solvent, following the process by time‐resolved synchrotron X‐ray radiation (**Figure** **5**). The authors suggested that when the medium surrounding the cellulose molecules becomes energetically unfavorable for molecular dispersion, regeneration starts and the initial process would consist in stacking the hydrophobic glucopyranoside rings (driven by hydrophobic interactions) to form monomolecular sheets, which then would line up by hydrogen bonding to form Na–cellulose IV type crystallites, a hydrate form of cellulose II. This constitutes the first experimental evidence of the development of hydrophobically stacked monomolecular sheets which has been hypothesized first by Hermans and later by Hayashi.^[^ ^137^, ^138^ ^]^ Later, the theoretical work of Miyamoto et al.^[^ ^139^ ^]^ simulated the regeneration of cellulose by MD, supporting the hypothesis of Hermans and Hayashi.

Among other interesting results, it was recently found that the water contact angle of regenerated cellulose films increases with lower water solubility of the coagulant. Most likely, this is due to cellulose amphiphilicity where the exposition of the hydrophobic areas to a polar environment is energetically unfavorable, thus leading to the reorientation of the more hydrophilic parts of cellulose (OH groups) toward the film interface.^[^ ^140^ ^]^

This study elucidates cellulose self‐assembly mechanisms, revealing that both hydrogen bonding and hydrophobic stacking (notably along the (110) plane) govern nanocrystal regeneration. Synchrotron XRD confirms a transition from disordered to ordered structures via hydrophobic (110) and hydrogen‐bonded (020) planes. The use of 5 wt.% Na_2_SO_4_ and timed regeneration show staged structural evolution. Despite clear evidence for urea‐assisted regeneration, long‐term stability in diverse environments remains unclear. Future work should clarify solvent‐specific effects and enable scalable, directional assembly strategies for advanced cellulose‐based materials.

#### 2.3.3 Aqueous Counter Collision

**Figure 6:**
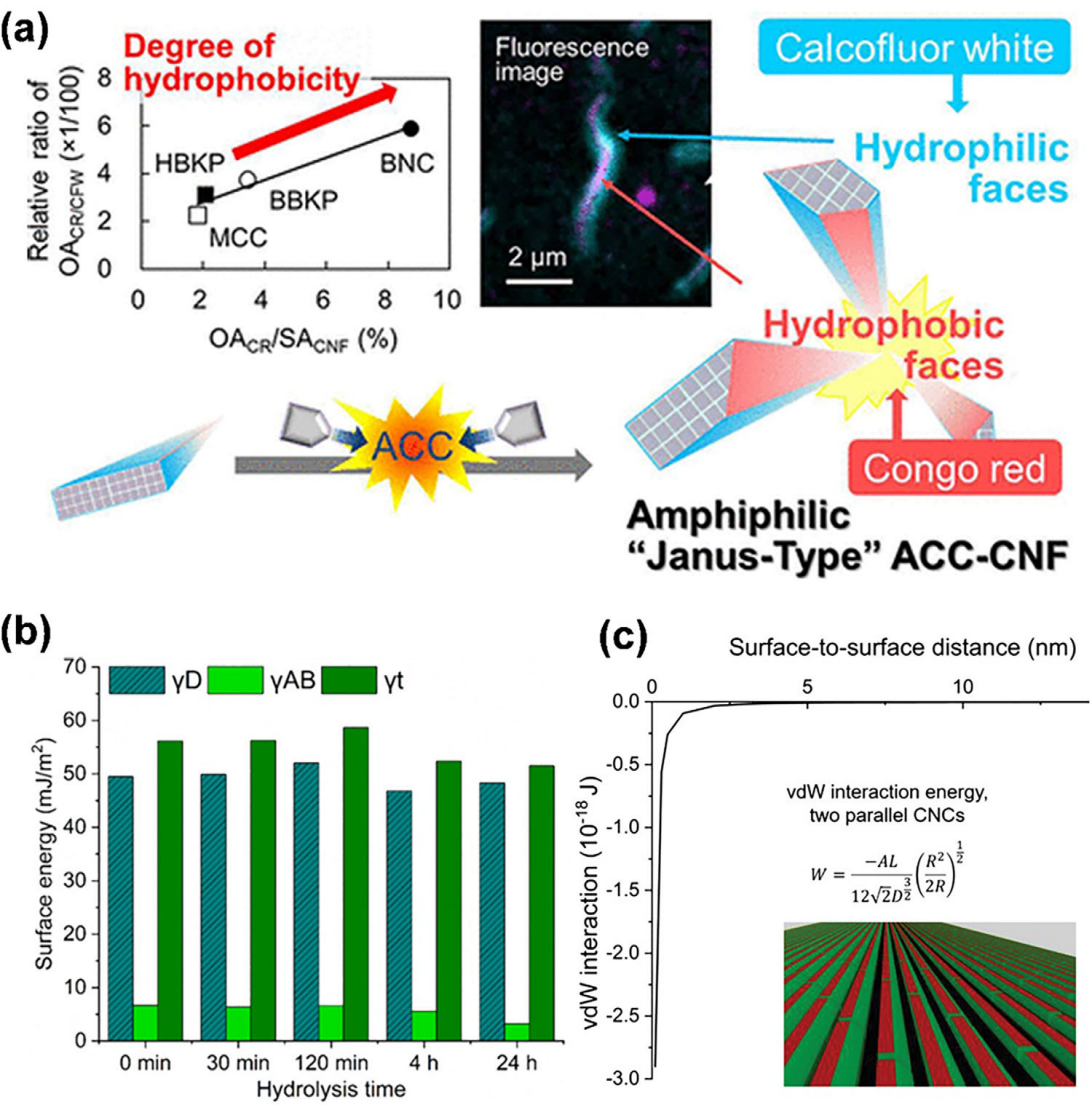
a) The principle of ACC technology. Simultaneously, confocal laser scanning microscope images of ACC‐BNC were also presented. The dye adsorption area is utilized to assess the extent of hydrophobicity. Here, OA_CR_ and SA_CNF_ represent the occupied area of Congo Red and the surface area of CNF, respectively.^[^ ^32^ ^]^ b) Inverse gas chromatography results for the total surface energy (γt) and its dispersive (γD) and acid‐base (γAB) components in 0% RH.^[^ ^142^ ^]^ c) The feasibility for regulating stacking modes is provided by the relationship between van der Waals interaction energy and the distance between amphiphilic planes.^[^ ^142^ ^]^ Reprinted with permission from ref.[33] Copyright: 2021, ACS; Reprinted with permission from ref.[142] Copyright: 2021, Wiley.

The recent research conducted by Tsuji et al. will serve as a crucial reference and offer novel insights for the advancement of nanotechnology.^[^ ^31^, ^32^ ^]^ They pioneered a novel method for reducing natural cellulose fibers to nanofibers through aqueous counter collision (ACC). The cellulose suspension is sprayed from dual nozzles and undergoes collision, peeling off the amphiphilic surfaces of cellulose under high speed and pressure. The preparation process and characterization data are depicted in **Figure** **6**a. The surface characteristics of ACC‐CNF were examined using Congo red as a probe for hydrophobic planes and calcium fluorescent white for hydrophilic planes, employing confocal laser scanning microscopy.^[^ ^141^ ^]^ A confocal laser scanning image is provided, clearly depicting independent hydrophilic and hydrophobic segments, where blue indicates hydrophilicity and red indicates hydrophobicity. Utilizing a function based on the adsorbed dye area and the apparent area of cellulose nanocrystals to assess hydrophobicity demonstrated a higher degree of exposure to hydrophobic surfaces, thereby validating the amphiphilic Janus‐type structure.

Solala et al.^[^ ^142^ ^]^ observed that the aggregation of hydrolyzed CNCs can result in a reduction in the pore size distribution of the original fibers. Hence, the investigation focused on the relationship between van der Waals interactions among CNCs and the nanoscale stacking of cellulose.^[^ ^133^ ^]^ CNC suspension can generate diverse solid components through evaporation or freezing, with the crucial aspect being the utilization of the amphiphilicity of cellulose crystals.^[^ ^143^ ^]^ Inverse gas chromatography was employed to unveil structural rearrangement and changes in the total surface energy, particularly a slight decrease in its acid‐base components, after 4 hours of hydrolysis. (Figure 6b) Due to the proximity of shortened cellulose fragments, they now have more degrees of freedom, leading to fewer obstacles in assembling into a more energetically favorable state. There is a slight structural change within the van der Waals forces, occurring at a scale of a few nanometers. At a surface distance of less than 2 nm, the van der Waals attraction is significantly stronger than at a distance greater than 5 nm. (Figure 6c) The surface distance between microfibers ranges from 4.6 to 1.6 nm, suggesting that the significant van der Waals attraction provides a reasonable explanation for the observed nanoscale stacking.^[^ ^142^ ^]^

This study provides an in‐depth exploration of the driving forces, including hydrogen bonding, hydrophobic interactions, and aqueous counter collision, in the regulation of the microscopic Janus structure of CNMs. While it successfully reveals various interaction mechanisms, it falls short in addressing the long‐term stability of these mechanisms in practical applications. Future research could further investigate the combined effects of different driving forces.

### 2.4 Janus‐Like CNCs in Crystalline/Amorphous Regions

**Figure 7:**
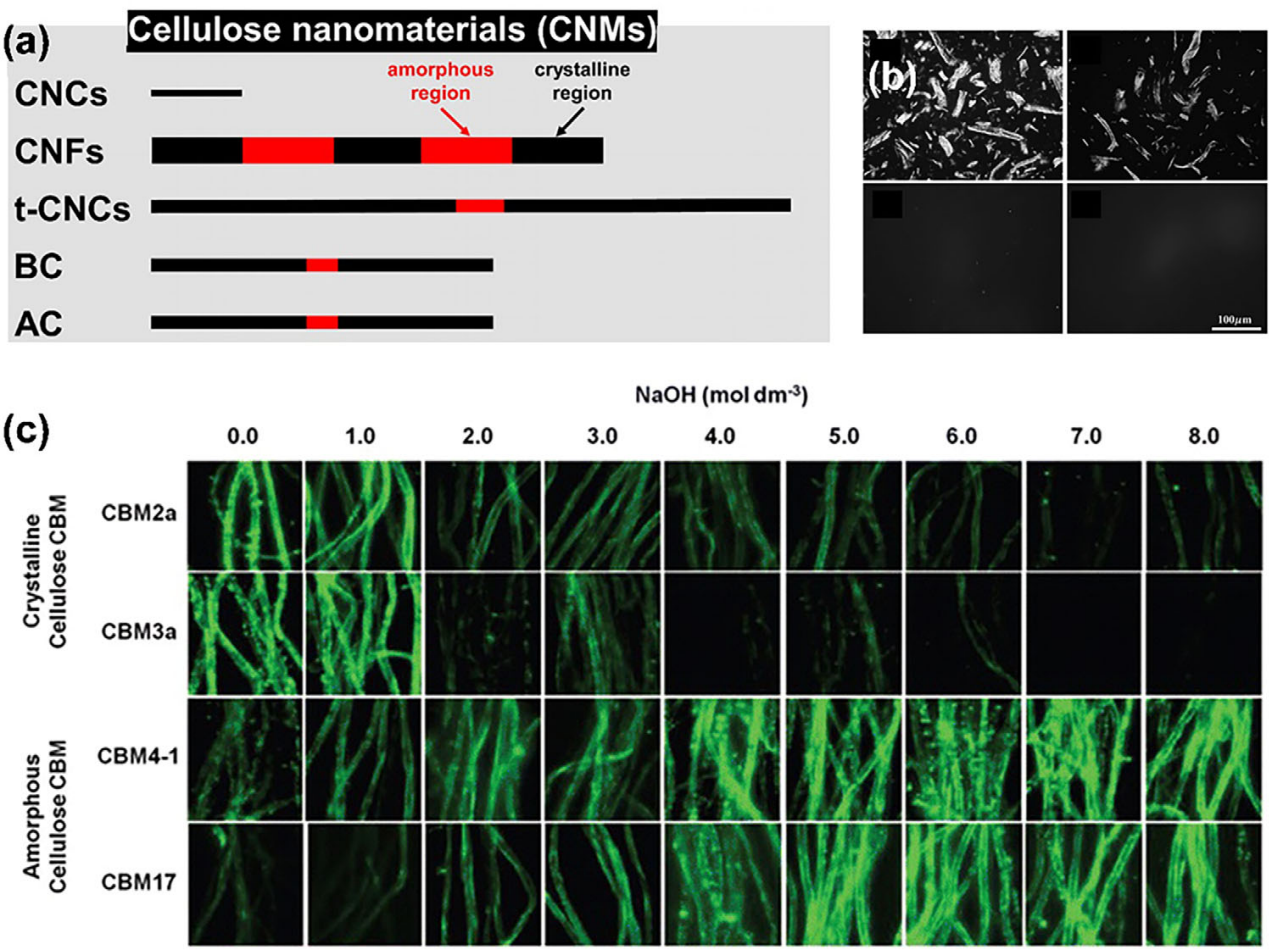
a) Comparison of the size and distribution of the crystalline/amorphous region of CNMs.^[^ ^150^ ^]^ b) Polarization Microscopic Images of Crystal to Amorphous Transformation of Cellulose in ethylenediamine/NaSCN.^[^ ^147^ ^]^ c) Fluorescence imaging of four types of CBM combined with crystalline and amorphous regions after treatment with a certain concentration of NaOH (0–8 m).^[^ ^149^ ^]^ Reprinted with permission from ref.[147] Copyright: 2016, ACS; Reprinted with permission from ref.[149] Copyright: 2023, ACS; Reprinted with permission from ref.[150] Copyright: 2023, ACS.

Previous studies have found that the molecular chain arrangement in the crystalline region is regular, and the density is higher than that in the amorphous region. The refractive index of the crystalline region and the amorphous region in the polymer is different, and the higher the crystallinity, the better the transparency. By studying the crystallinity of cellulose nanomaterials, we can regulate the physical and chemical properties of cellulose.^[^ ^144^, ^145^ ^]^ In order to better reflect the comparison of the size and distribution of the crystal/amorphous region, we provide a more clear and intuitive display as shown in **Figure** **7**a. Due to the limited number of amorphous regions in CNC, it is very suitable for studying the amphiphilicity of crystal planes. Different volumes of amorphous regions can affect the size of hydrophobic and hydrophilic areas and the formation of Janus.

The information of the crystalline and amorphous regions of cellulose can be characterized using X‐ray diffraction (XRD). The preparation of amorphous cellulose is generally achieved by dry grinding the cellulose in a ball mill, or by treating the cellulose with a concentrated (18–20%) sodium hydroxide solution.^[^ ^128^, ^146^ ^]^ Hattori et al.^[^ ^147^ ^]^ prepared amorphous cellulose using amine/inorganic salt solvents. Figure 7b shows the polarization microscopic images of cellulose crystals gradually transforming into amorphous over time. We can see that the amorphous transformation of cellulose generally occurs from the outside to the inside. However, neither alkali treatment nor liquid ammonia, amine, or ethylenediamine treatment will result in complete amorphization of cellulose.

In order to visually analyze the amorphous process of cellulose and to further investigate the transformation and regulation of the crystalline and amorphous regions of CNMs. Kljun et al.^[^ ^148^ ^]^ used carbohydrate‐binding modules (CBMs) to assess changes in cellulose crystallinity during the mercerization process of cotton fibers. The authors used two type A and two type B CBMs coupled with fluorescence imaging to visualize the changes in the crystallinity at various stages of the mercerization process, which agreed with the results obtained from traditional XRD and FTIR techniques as represented in Figure 7c. CBMs can be classified into 68 families and based on their three‐dimensional structures and activity, they are classified as type A, type B, and type C. Type A CBMs are surface‐binding type proteins with a greater affinity toward crystalline cellulose. Unlike type A, type B proteins show affinity toward the amorphous cellulosic regions by binding to glycan chains. Yet, type B CBMs cannot recognize glycan chains consisting of less than three sugar units while type C CBMs exhibit binding specificity for 1–3 sugar units.^[^ ^149^ ^]^ We will provide a detailed explanation in the hairy Janus section, which can also provide a basis for us to choose suitable CNMs to construct Janus materials.

**Figure 8:**
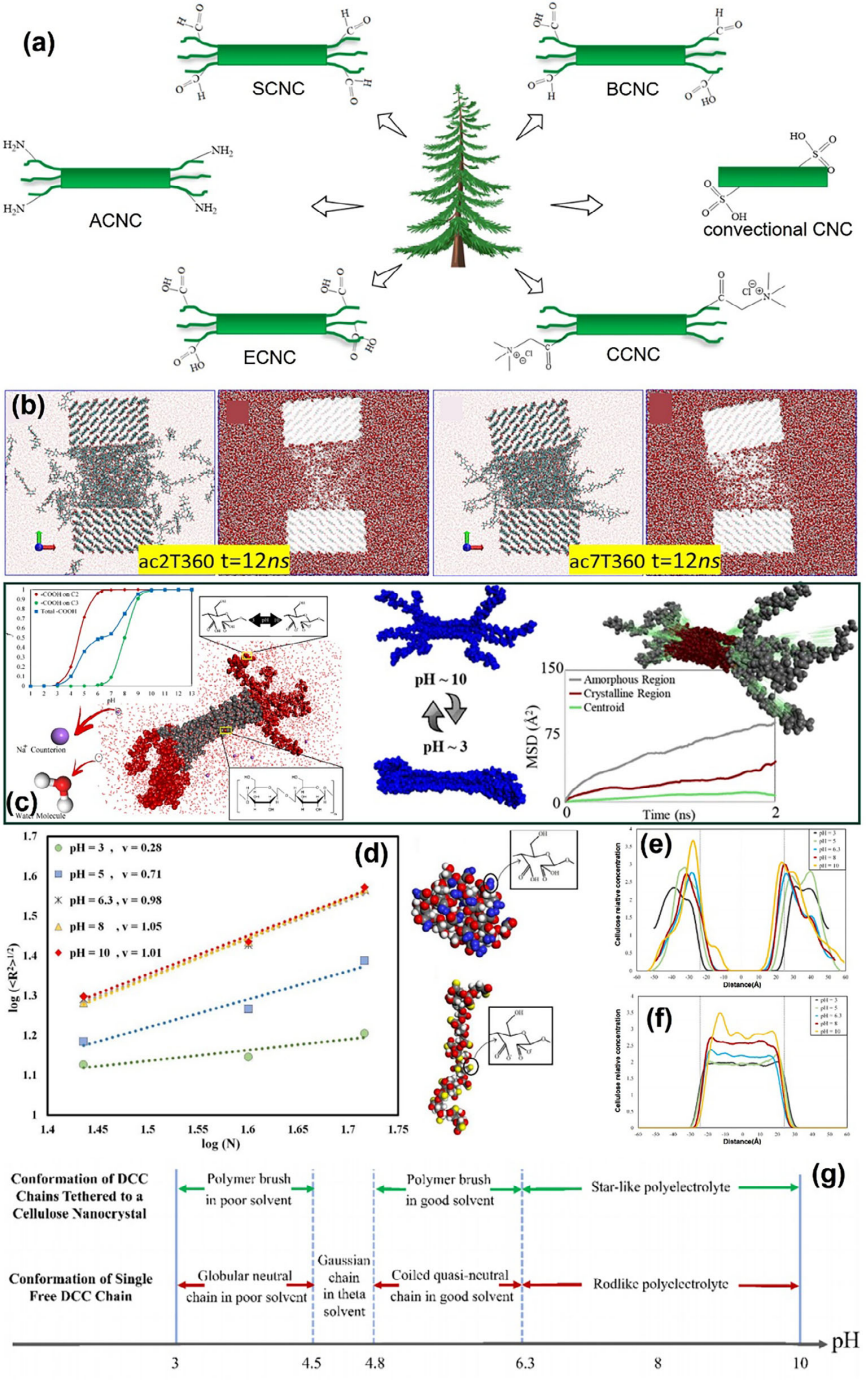
a) Types of convectional and hairy nanocelluloses: Carboxyl groups electrosterically stabilized cellulose nanocrystalline (ECNC), aldehyde groups sterically stabilized cellulose nanocrystalline (SCNC), aldehyde‐carboxyl groups bifunctional cellulose nanocrystalline (BCNC), quaternary ammonium groups cationic cellulose nanocrystalline (CCNC) and primary amine groups cellulose nanocrystalline (ACNC).^[^ ^154^ ^]^ b) Simulation diagram of nanocellulose dissolution in water. ac2T/7T360 indicates that the chain length of the amorphous region is 2 cellobiose units (4 glucose rings) /7 cellobiose units and the temperature is 360 K. c) The ECNC particle model is used to perform constant pH molecular dynamics (CpHMD) simulations.^[^ ^155^ ^]^ d) Variation of polymer size with pH. Flory exponent is 0.29 at pH ≈ 3, 0.74 at pH ≈ 5 and ≈ 1 at pH > 6.3. Carboxyl molecular models for protonation and deprotonation. e) Concentration distribution of ECNC amorphous chain. f) Concentration distribution of ECNC crystal chain. g) Conformational preference of single‐free and tethered dicarboxylated cellulose (DCC) chains over different pH.^[^ ^156^ ^]^ Reprinted with permission from ref.[154] Copyright: 2021, Elsevier; Reprinted with permission from ref.[155] Copyright: 2021, ACS; Reprinted with permission from ref.[156] Copyright: 2019, ACS.

The acid has different degrees of hydrolysis to the crystalline and amorphous regions of cellulose, so the cellulose nanocrystalline materials with higher crystallinity can be obtained.^[^ ^151^ ^]^ Controlled oxidation of cellulose fibrils causes partial disintegration of amorphous chains to obtain Janus hairy nanocelluloses (HNCs), which have amorphous areas (hair) at both ends and crystalline areas in the middle. **Figure** **8**a shows a variety of HNCs currently available.^[^ ^152^, ^153^, ^154^ ^]^ Considering the change of H^+^ ions in the solution, the change of pH will affect the morphology of cellulose nanomaterials and also affect the colloidal properties. Therefore, we summarize the pH response of cellulose nanomaterials in controlled expansion and diffusion. The dissolution of cellulose nanomaterials in water depends on the chain length of the amorphous region. When the temperature is 360 K and the time is 12 ns, the shorter chain length is easier to separate (Figure 8b). Wang et al. proposed that the dissolution mode in the amorphous region belongs to the single‐chain dissolution, chemical (interchain hydrogen bonding), and physical (chains intertwining with each other) dissolution synergistic mechanism, which is related to the hydrogen bond and chain length kinetics between cellulose−cellulose (C−C) and cellulose−water (C−W).^[^ ^155^ ^]^ When the concentration of H^+^ ions in aqueous solution changes, Youssefian et al.^[^ ^156^ ^]^ studied that carboxylated trichoid ECNC exhibits a specific reaction under different pH conditions, and the protonation state of carboxyl groups can affect the nanostructure of ECNC (Figure 8c). The amorphous region studied is dicarboxylated cellulose (DCC). For ECNC with only a carboxyl group, the microscopic state can be ignored and only the macroscopic state of protonation can be considered. Constant pH molecular dynamics simulation (CpHMD) was used to simulate the effect of pH on ENCC.^[^ ^157^ ^]^

There are two adjacent carboxyl groups on C2 and C3 of DCC chains. Since the pKa of the carboxyl group depends on how much hydroxyl bond is polarized, carboxyl groups on C2 have different proton affinity than the ones on C3. The fraction of the charged monomers (ƒ) can be calculated by the Henderson‐Hasselbalch function.^[^ ^158^, ^159^ ^]^

(1) $$N_{D}=\left(1-\frac{10^{pK_{a1}-\mathrm{pH}}}{1+10^{pK_{a1}-\mathrm{pH}}}\right)N_{2}+\left(1-\frac{10^{pK_{a2}-\mathrm{pH}}}{1+10^{pK_{a2}-\mathrm{pH}}}\right)N_{3}$$

where N_D_, N_2_and N_3_ are the numbers of deprotonated sites and total carboxyl groups on C2 and C3, respectively (pK_a1_ ≈ 4.6 and pK_a2_ ≈ 8.0).^[^ ^160^ ^]^

Figure 8c shows the change of ƒ with the increase of pH calculated by the Henderson‐Hasselbalch function. The deprotonation of carboxyl groups on C2 occurs between pH ≈ 3 and 6. For C3, deprotonation occurs at pH between 6 and 10. The resulting deprotonation state of ENCC varies between pH ≈ 3 and 10. According to mean‐squared displacement (MSD), the particle displacement in amorphous region is longer than that in the crystalline region. At the same time, the diffusion coefficient $D_{\mathrm{sim}}=\frac{1}{6}\;\lim_{t\to \infty }\frac{d(\mathrm{MSD})}{\mathrm{dt}}$ is calculated by adding the MSD value.^[^ ^156^ ^]^ The changes of the hydrodynamic properties of ENCC nanoparticles under different pH conditions were studied. The diffusion constant of amorphous chain increases with the increase of pH. In order to further study the effect of pH change on amorphous region, the solubility of water as solvent at different pH values was investigated based on Flory exponent (v).^[^ ^161^ ^]^ When water is a poor solvent at low pH, the DCC chain adopts a collapse conformation (Figure 8d). By studying the distance from the particle to the crystal region as pH increases, the concentration distribution in Figure 8e moves toward the center of ENCC, indicating that the amorphous region is swollen, while the crystal region length in Figure 8f shrinks. Figure 8g summarizes that as pH increases from ≈3 to ≈10, the nature of DCC chains undergoes a major transformation from polymer to quasi‐neutral polymer, and to polyelectrolyte. This variation of DCC nature that is stemmed from increasing fraction of charged monomers significantly affects on its conformation and thus the nanostructure of the ENCC.^[^ ^156^ ^]^

Janus cellulose nanomaterials exhibit dual structural and functional asymmetry, achieved through crystal/amorphous interface modulation, selective surface modification, and pH‐responsive behavior. Molecular simulations elucidate the underlying mechanisms, such as hydrogen bonding networks and conformational dynamics, offering theoretical insights for designing smart interfaces. Moving forward, research efforts should prioritize precise interfacial functionalization, multiscale computational modeling, and controlled fabrication techniques to advance applications in targeted delivery, programmable self‐assembly, and sustainable material engineering.

## 3 Construction of Janus Materials via Chemical Modification of CNMs

### 3.1 Reaction Sites between the Reducing End and Surface Groups of CNMs

Theoretical values of all hydroxyl groups on cellulose molecules exposed to the environment: Taking completely dry CNC as an example, the average relative molecular weight of (C_6_H_10_O_5_)_n_ anhydro glucopyranose units is 162.14 g mol^−1^. The hydroxyl content should be at least greater than 6.177 × n mmol g^−1^ (n is the degree of polymerization of the molecular chain, which is an uncertain value).^[^ ^162^ ^]^ The determined amount of available aldehyde groups and surface hydroxyl groups of CNCs are summarized in **Table** **1**. The amount of total hydroxyl groups on the CNC surface can vary greatly with the cellulosic sources and crystal dimensions, ranging from 0.955 to 2.646 mmol g^−1^ (Table 1). Note that the content of sulfate esters on the CNC surface derived from sulfuric acid hydrolysis is commonly reported within the range of 0.08‐0.35 mmol g^−1^.^[^ ^163^, ^164^ ^]^ The number of terminal aldehyde groups of CNCs can be determined via direct and indirect measurements, with the reported values ranging from 0.018 to 0.0738 mmol g^−1^ (Table 1). According to the reported results, the number of hydroxyl groups on the surface of CNCs is approximately 50–150 times that of the terminal aldehyde group.

**Table 1:** Reported quantities of terminal aldehyde groups and surface hydroxyl groups of CNCs from various sources.^[^ ^165^ ^]^

Active group	Cellulose Source	Content of group [mmol g^−1^]	Measurement techniques
**Aldehyde group**	Whatman filter paper	0.035–0.045	‐
	Cotton linter	0.0548–0.0738	Formazan method
	wood pulp	0.018	Colorimetric assays
	CelluForce product	0.0245	Hydroxylamine
	Cotton linters	0.0249	Fehling test
**Hydroxyl group**	Cotton	0.955–2.646	Theoretical calculation
	Whatman filter paper	1.57–1.82	Theoretical calculation

### 3.2 Simple and Efficient Side‐Chain Modifications for Preparing Janus CNMs

**Figure 9:**
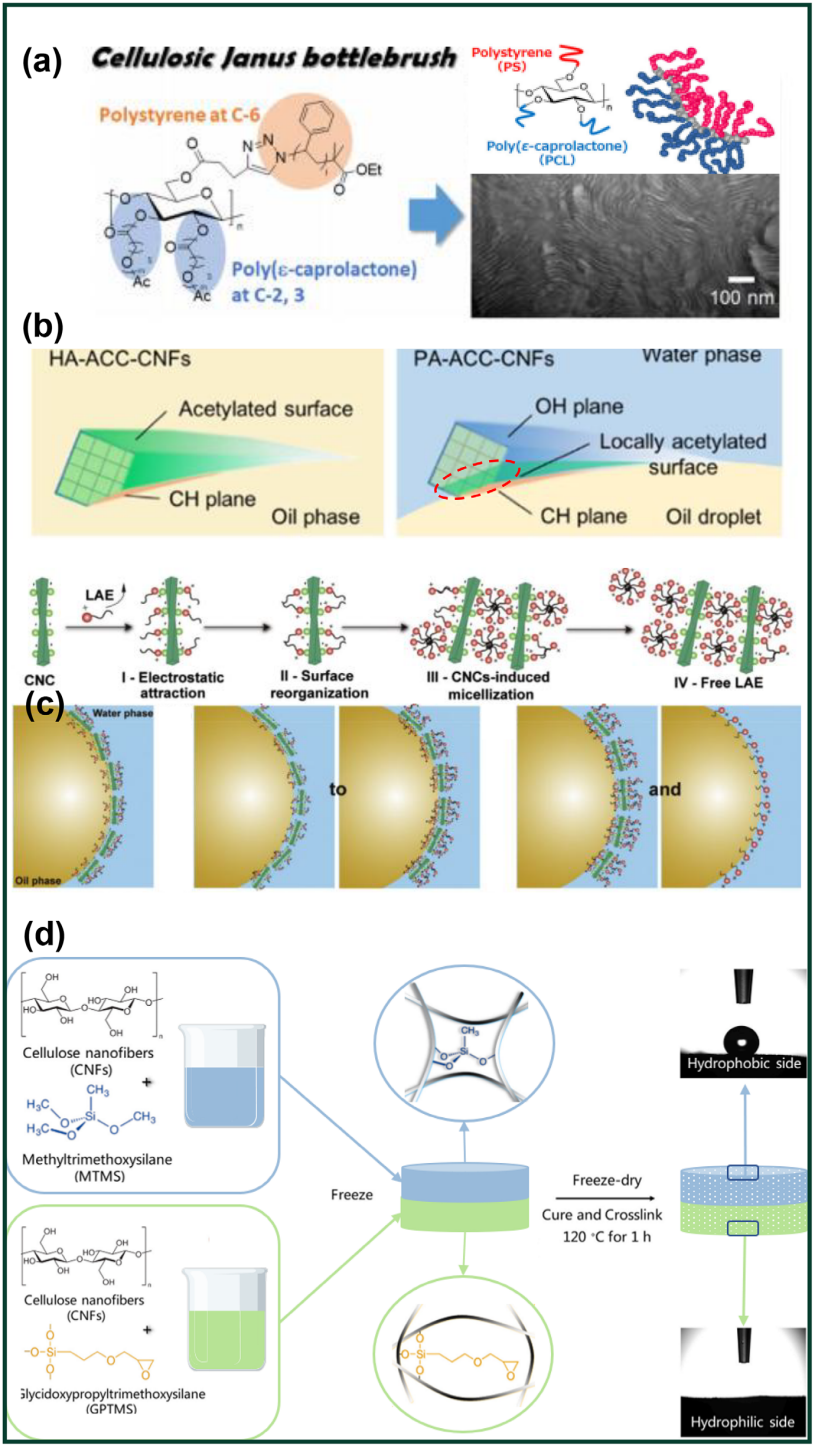
a) Schematic of regioselectively grafted cellulosic Janus bottlebrush fabrication.^[^ ^166^ ^]^ b) Schematic illustrations of surface acetylation in the homogeneous dispersion system (left) and the Pickering emulsion (PE) system (right).^[^ ^167^ ^]^ c) Schematic illustration showing of four‐stage complex formation as a function of LAE concentration in CNC suspension.^[^ ^101^ ^]^ d) Preparation of Janus hybrid sustainable CNF sponge via silanized cellulose.^[^ ^69^ ^]^ Reprinted with permission from ref.[167] Copyright: 2021, Elsevier; Reprinted with permission from ref.[69] Copyright: 2021, Elsevier; Reprinted with permission from ref.[166] Copyright: 2021, Springer; Reprinted with permission from ref.[101] Copyright: 2021, Elsevier.

The Hydroxyl group has numerous modification conditions, and by itself can improve hydrophilicity, increase plasticity, and improve durability and adsorption capacity of cellulose. Sakakibara et al.^[^ ^166^ ^]^ reported the synthesis and self‐assembly of a new Janus bottle brush using cellulose as the polymer backbone. Branches containing poly(ε‐caprolactone) and polystyrene were inserted into anhydrous glucose units C‐2,3 and C‐6, respectively (**Figure** **9**a). Adding functional groups is conducive to the construction of Janus materials to achieve intelligent functions such as reaction, and the easiest way to achieve the production of Janus materials is with a one‐step modification. Nypelö et al.^[^ ^30^ ^]^ previously reported that periodate oxidation and ozone pretreatment on both sides of cellulose films could render cellulose films specifically functional.

The ACC technique in Section 2.3.3 has been shown to expose the hydrophobic surfaces of cellulose nanomaterials, enabling the isolation of a single hydrophobic plane. This method has gained significant attention for Janus cellulose material preparation. The Tetsuo Kondo team later proposed a practical modification approach, providing valuable insights for further research.^[^ ^32^, ^33^, ^167^ ^]^ In their study, amphiphilic cellulose nanofibers (ACC‐CNFs) were modified via two acetylation methods. The first involved dispersing ACC‐CNFs in an oil/water Pickering emulsion, followed by acetylation with acetic anhydride and other reagents (PA‐ACC‐CNFs). The second method used solvent exchange into N, N‐dimethylacetamide, with acetylation carried out for 30 minutes (HA‐ACC‐CNFs).^[^ ^167^ ^]^ ACC's ability to expose the hydrophobic plane of CNFs enhanced their stability in emulsion, outperforming methods like high‐pressure homogenization (Figure 9b).^[^ ^33^ ^]^ It is particularly noteworthy that PA‐ACC‐CNFs display environmentally responsive self‐aggregation during the drying process. The locally acetylated ACC‐CNFs spontaneously aggregate at the air/water and glass/water interfaces. When thin films are cast from aqueous suspensions, these materials form Janus‐like structures with anisotropic surfaces, exhibiting unique surface properties tied to their assembly behavior.

Another common strategy is to link non‐polar to CNC to improve emulsification capacity, while the addition of ionic surfactants can also enhance the electrostatic interaction of CNMs For example, lauric arginate ester (LAE), a cationic small molecule surfactant, is used to regulate the behavior of the CNC at the sunflower oil/water interface.^[^ ^100^, ^168^ ^]^ The assembly of CNC in the aqueous phase is highly dependent on the LAE concentration, and emulsions with different stable behaviors can be produced at different cationic surfactant levels (Figure 9c). At lower LAE levels, the droplets were stabilized by a complex containing partially neutralized CNC. With the increase of LAE concentration, the emulsification and stabilization ability was enhanced, and the Pickering emulsion obtained was also more resistant to wrinkling and agglomeration after long‐term storage, indicating that CNC modification of surfactants is also an effective way to obtain emulsions.

**Figure 10:**
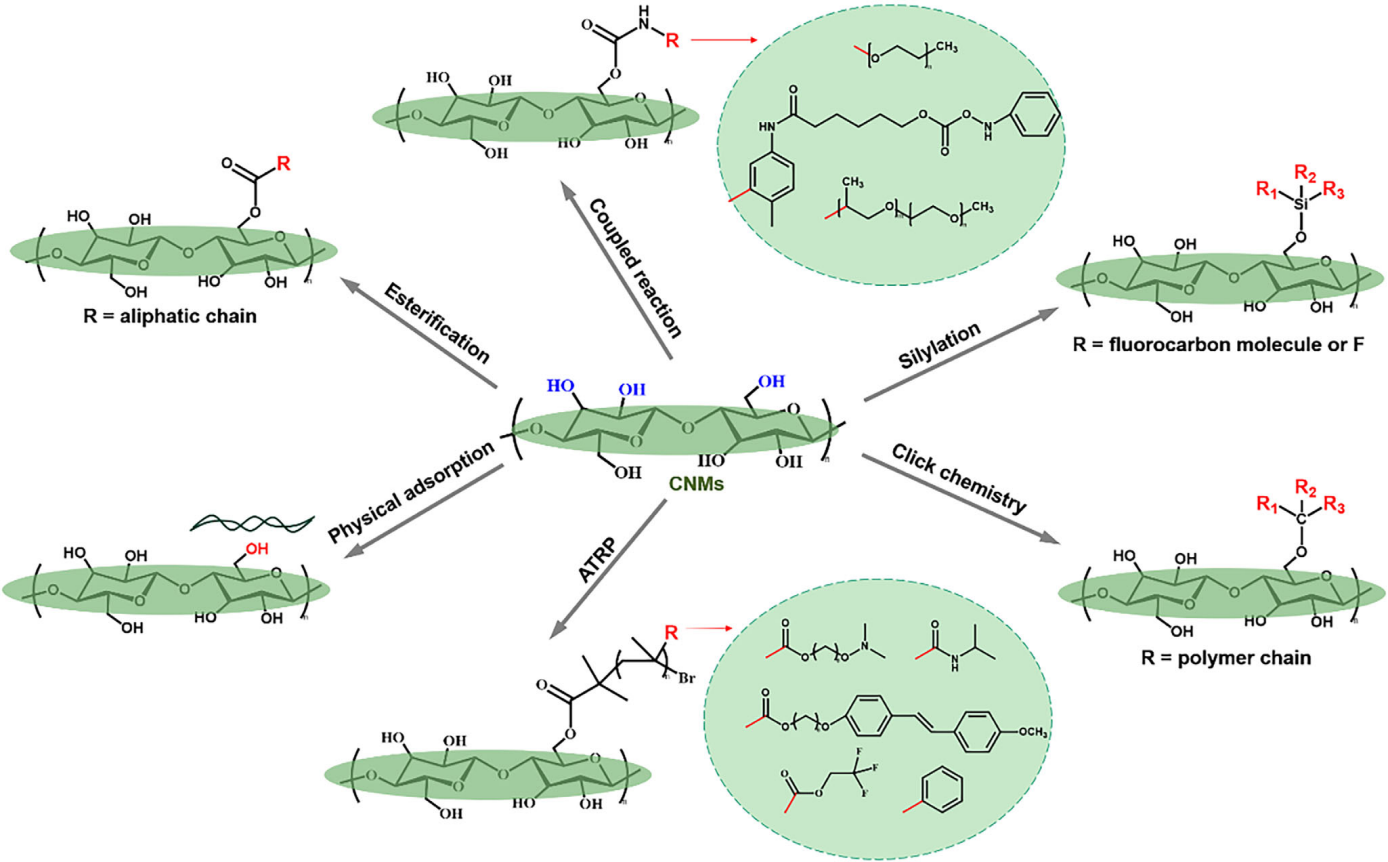
Several common hydrophobic modification methods for cellulose nanomaterials and recommended functional groups.^[^ ^169^, ^170^, ^171^, ^172^ ^]^

The Janus CNMs discussed above are primarily obtained through hierarchical modification of cellulose molecular chains, a key approach in Janus CNM research. In Section 1.2 and Figure 2, we highlighted that over 80% of Janus cellulose materials are composites. Figure 9d illustrates the creation of composite Janus CNMs through hydrophobic modification.^[^ ^69^ ^]^ Briefly, the CNF Janus hybrid sponge was fabricated by freeze‐drying of two separate CNF suspensions into one, each prepared separately by introducing CNFs in methyltrimethoxysilane or 3‐glycidoxypropyltrimethoxysilane for hydrophobic or hydrophilic performance, respectively. The sponge demonstrated satisfactory mechanical stability with an excellent recovery from 80% compressive strain and high pore tortuosity. When employed for oil‐water separation, the Janus hybrid sponge could selectively be used to collect water or oil by just switching its side facing the oil‐water mixture feed via unidirectional gravity‐assisted separation, with recyclability. The fabrication of such Janus hybrid sponge is one of the many approaches for utilizing nanofibers in structurally adaptive, self‐supported asymmetric membrane structures in a 3D network. **Figure** **10** summarizes various efficient methods and functional groups for hydrophobic modification of CNMs, providing valuable reference for researchers in Janus CNM development.

### 3.3 Architecture by Reduction End Modified Janus

**Figure 11:**
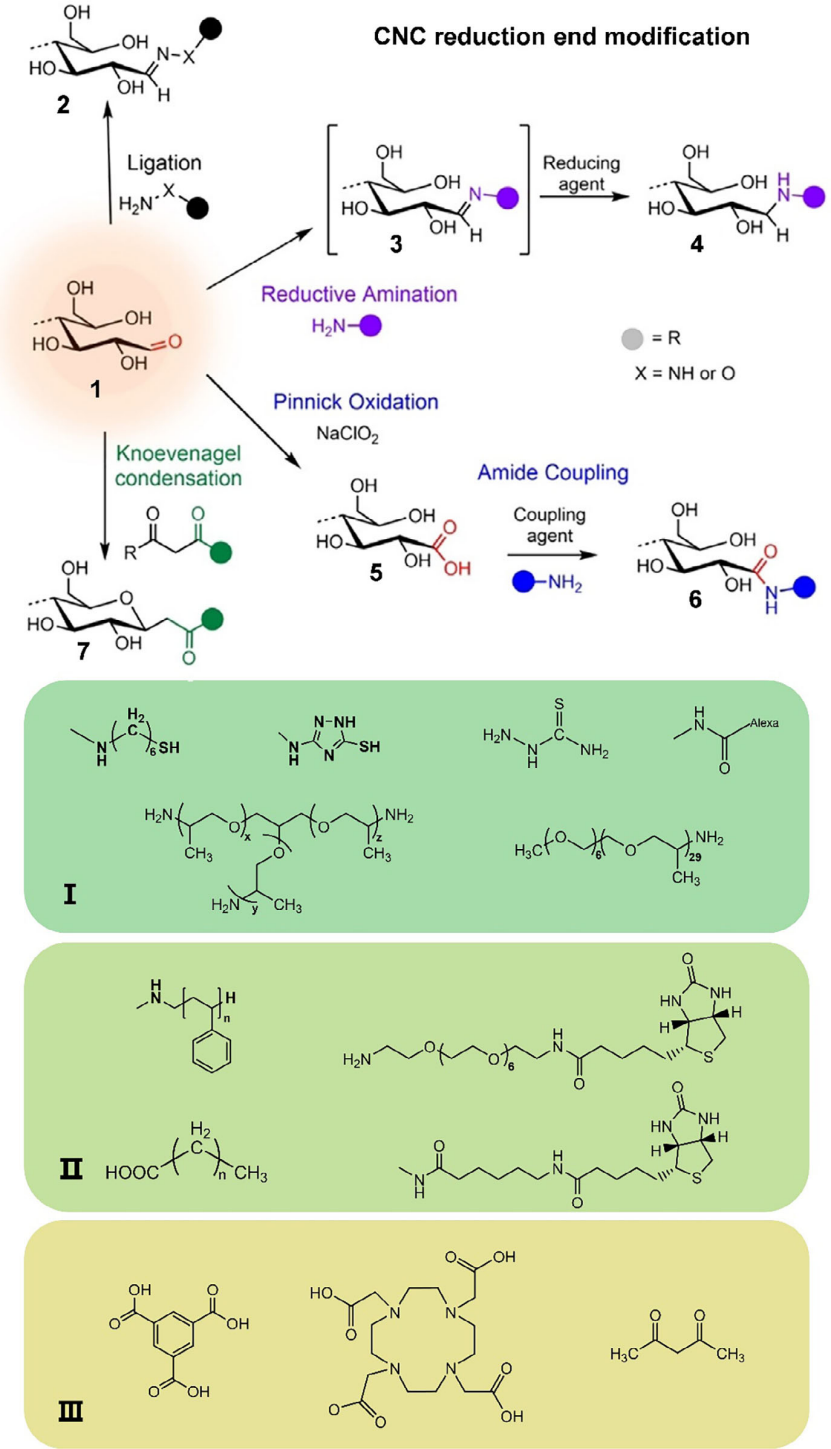
Ligation of hydrazine analogues (NH_2_‐NH‐R) or hydroxylamine derivatives. (NH_2_‐O‐R) affording hydrazones or oximes (2). reductive amination forming an imine intermediary (3) that is reduced to a stabilized secondary amine (4). Pinnick oxidation affording carboxylated REGs (5) that allow for subsequent amidation (6). Knoevenagel condensation provides C‐glycoside ketone (7). I Responsive Janus group (Au, temperature, fluorescence).^[^ ^38^, ^52^, ^165^, ^176^ ^]^ II Functional Janus groups (amphiphilic, electrical, supramolecular assembly).^[^ ^165^, ^174^, ^177^ ^]^ III Active site expansion group.^[^ ^165^ ^]^ Reprinted with permission from ref.[28] Copyright: 2013, ACS; Reprinted with permission from ref.[52] Copyright: 2019, ACS; Reprinted with permission from ref.[165] Copyright: 2016, RSC; Reprinted with permission from ref.[174] Copyright: 2018, ACS; Reprinted with permission from ref.[176] Copyright: 2019, ACS; Reprinted with permission from ref.[177] Copyright: 2018, ACS.

Most synthetic approaches toward modifying the REGs of cellulosic substrates rely on aldehyde‐specific chemistry.^[^ ^10^ ^]^ We have compiled the modification techniques and reaction conditions employed in the study of constructing Janus materials through the reducing end (**Figure** **11** and **Table** **2**). In this Review, we classify them as hydrazine, hydroxylamine, and thiosemicarbazide ligation (**2**), reductive amination (**3/4**), Pinnick oxidation (**5**) followed by amidation (**6**), and Knoevenagel condensation (**7**). These reactions are mostly used as the initial step to activate the anomeric center for, e.g., subsequent polymer grafting or click chemistry.

**Table 2:** Typical modification reaction conditions at the reducing end.

Reaction type	Reactants	Medium, pH conditions/catalysis	Temperature, duration
ligation^[^ ^178^, ^179^, ^180^, ^181^ ^]^	aliphatic/aromatic hydrazine analogs	aqueous, (mostly) alkaline	25–35 °C, 24–72 h
		DMSO (anhydrous), Ar atmosphere	50–60 °C, 4 days
	hydroxylamines	aqueous, acidic, or DMAc/LiCl (2.5% w/v), Et3N	40 °C, up to 700 h
	thiosemicarbazide	aqueous, acidic	60–65 °C, 90 min
reductive amination^[^ ^176^, ^177^, ^182^, ^183^ ^]^	1° amine ligands + reducing agent	aqueous buffer, acidic or alkaline	70 °C, 24–72 h
		MeOH/acetic acid	50 °C, 12–16 h
		DMF	RT or 70 °C, 1 or 3 days
		DMAc/LiCl	70 °C, 24 h
Pinnick oxidation^[^ ^174^, ^184^, ^185^ ^]^	NaClO_2_	aqueous, acidic	RT, 20–48 h
Knoevenagel condensation^[^ ^180^ ^]^	1,3‐diketones	aqueous bicarbonate, pH 8.5	80–90 °C, 4–48 h

By modifying the reducing end and precisely controlling the self‐assembly behavior of CNCs, customized molecules can be introduced, allowing for the design of end‐to‐end, star‐shaped, and brush‐shaped nanocrystals.^[^ ^38^, ^173^, ^174^, ^175^ ^]^ In Figure 11, we also depicted the functional groups employed in Janus construction. Subsequently, we will furnish a more intricate elucidation of the Janus construction process, with a specific emphasis on the cellulose reduction terminal.

**Figure 12:**
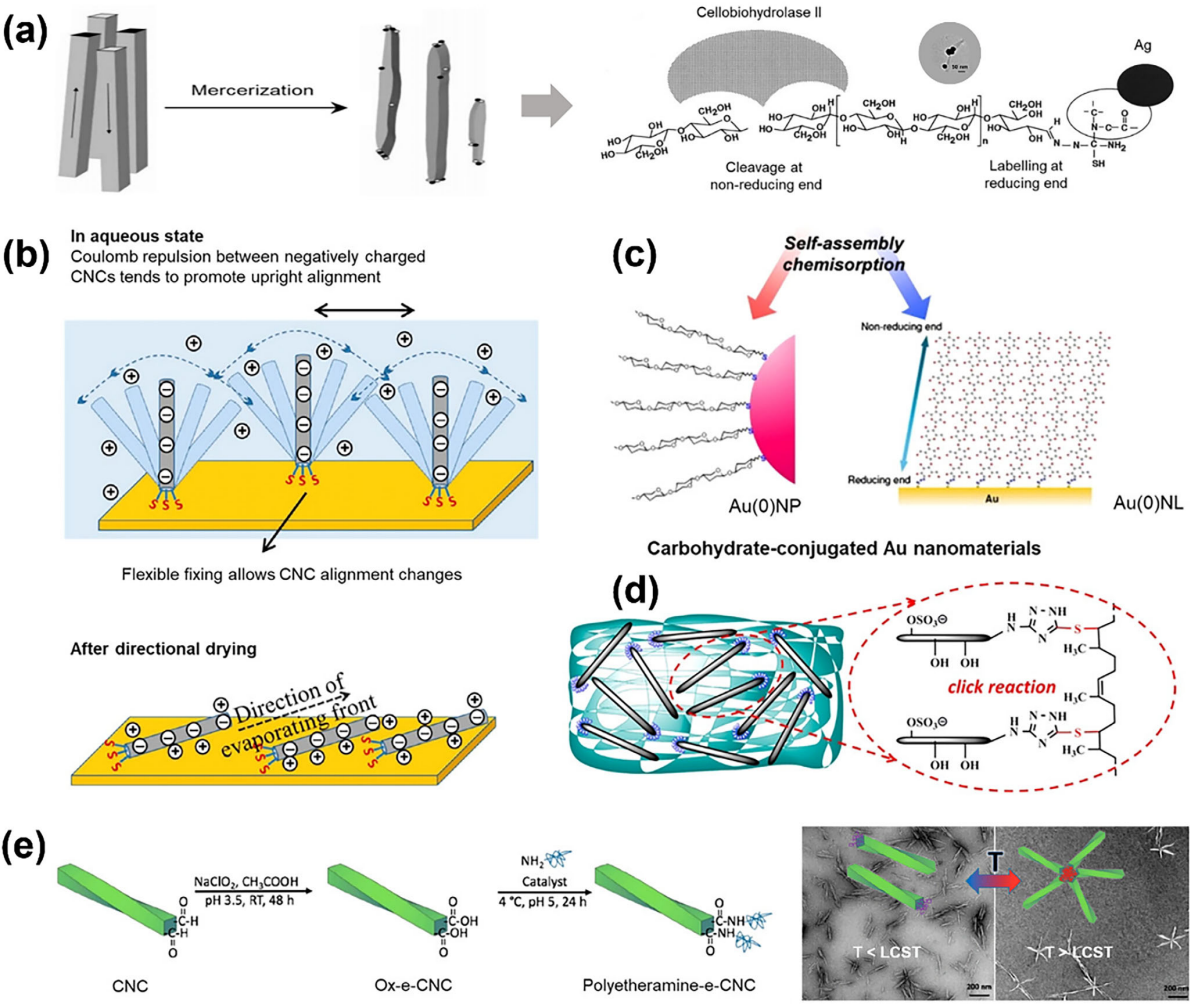
a) The effect of specific reduction end labeling with Ag^0^NPs.^[^ ^182^ ^]^ b) Representation of cilia‐mimetic surfaces based on SH‐functionalized CNCs chemisorbed to a gold substrate.^[^ ^38^ ^]^ c) SAMs of TSC‐modified cellulose following their spontaneous chemisorption to gold nanoparticles (Au^0^NP) or gold nanolayers (Au^0^NL).^[^ ^187^ ^]^ d) Reactive Compatibility of Reducing End Modified Nanocrystals with Natural Rubber.^[^ ^184^ ^]^ e) End‐functionalization of CNCs with polyetheramines and TEM images showing the temperature‐triggered formation of star‐shaped assemblies above the LCST of the polyether amine chains.^[^ ^52^ ^]^ Reprinted with permission from ref.[52] Copyright: 2019, ACS; Reprinted with permission from ref.[182] Copyright: 2006, ACS; Reprinted with permission from ref.[184] Copyright: 2018, ACS; Reprinted with permission from ref.[38] Copyright: 2013, ACS; Reprinted with permission from ref.[187] Copyright: 2010, Elsevier.

Hieta and co‐workers first observed the parallel orientation of the primary cellulose I chain in an algal species (Valonia), selectively oxidized by NaCIO_2_ and labeled reg with silver nanoparticles (Ag NPs).^[^ ^186^ ^]^ Kuga and Brown optimized this labeling approach for Ramie and bacterial cellulose using the aldehyde‐specific ligation of thiosemicarbazide (**Figure** **12**a).^[^ ^181^ ^]^ Most of the synthesis strategies for the reducing end of Janus cellulose are based on mature chemistry provided by aldehyde groups.^[^ ^10^ ^]^

Cellulose nanocrystals with specific adsorption of Au by thiol end groups or Ag by reductive amination can be used to prepare Janus materials with a capillary surface with orientation flow at one end and a fixation at the other end. It is of great significance for the directional creation of two‐dimensional Janus colloidal crystal materials. Lokanathan et al.^[^ ^38^ ^]^ were the first to translate the concept of cellulose–gold self‐assembled monolayers to CNCs, showing a directed assembly of end‐thiolated nanocrystals onto gold surfaces (Figure 12b). Kitaoka and co‐workers pioneered the exploitation of the inherent anisotropy of the cellulose chain for the design of functional, self‐assembled monolayers (Figure 12c). Their approach was both simple and trendsetting, using the spontaneous conjugation of topochemically thiolated cellulose to gold substrates.^[^ ^187^ ^]^ The conjugation of the derivative to gold resulted in a vectorial chain immobilization in which the parallel packing of the naturally occurring cellulose I crystal was replicated because of the topochemical thiolation. Thiolated methyl cellulose endowed the gold surfaces with thermally responsive wetting characteristics, on account of the reversible gelation behavior of the modified cellulose at temperatures above its lower critical solution temperature (LCST).^[^ ^188^ ^]^

Chemin et al.^[^ ^175^ ^]^ proposed a facile method to attach spheroidal dendritic macromolecules to the reduced ends of cellulose nanocrystals obtained from encapsulated animals (t‐CNC). The first four generations of polyamide dendrimers were studied to obtain hybrid t‐CNC. Hybrid t‐CNC combines the properties of rigid rod‐like nanocrystals and spherical and flexible PAMAM dendritic large crystals to form a hair‐like layer on the gold surface. Furthermore, the presence of an amino group induces a pH response characteristic of the hybrid t‐CNC, and the QCM‐D results show a reversible expansion/deexpansion behavior. A synthetic tree‐like functional bio‐based material was prepared and adsorbed onto gold to form a pH‐responsive Janus hair surface.^[^ ^7^, ^38^ ^]^

Based on the modification of the reduction end, the Ning team has carried out extensive research on the reduction end.^[^ ^189^, ^190^ ^]^ The strategy of triazole end‐grafting and long‐chain poly (ethylene oxide) modification at the CNC reduction end has significantly improved the redispersability and stability of nanocrystals in water due to the synergistic effect of spatial stability and electrostatic repulsion. The mechanical properties of the nanocomposite were found to gradually increase with the content of end‐functionalized CNCs (2.5–15 wt.%) and exceeded the reinforcement achieved with unmodified CNCs. For instance, the storage modulus (at 25 °C) increased from 1 MPa for the unmodified rubber to 12 MPa upon incorporation of 10 wt.% end‐modified CNCs (Figure 12d). In the region‐selective modification, we must consider the conditions that affect the content of and aldehyde groups and the success rate of the modification. In the work of Tan,^[^ ^7^ ^]^ the content of aldehyde groups varies between 7.65 and 46.30 µmol g^−1^ due to the different sources and manufacturing methods of the CNCs.

Cellulose can be used as a temperature‐responsive material modified by reducing end. The reduction end of CNCs is carboxylated and then peptide‐coupled with the primary amine of polyether amine. Each molecule has four binding sites, and a star‐shaped complex composed of four arms is constructed. This specific association is verified by changes in the mean diameter determined by dynamic light scattering and AFM. At temperatures above the LCST, the heat‐sensitive polymer chain on the reducing end of the CNCs collapses from hydrophilic to hydrophobic, and the interaction between the collapsed chains in water forms a star‐shaped assembly with 4, 5, or 6 reducing end modifications.

Lin et al.^[^ ^52^ ^]^ contributed to the specificity and control of the supramolecular assembly of multiple nanocrystals to generate star‐like Janus cellulose nanocrystals with a specific response to temperature, and proposed the reduction end‐graft of CNCs with thermally‐responsive polyetheramines in water suspensions when the temperature was increased to a lower critical solution temperature. The reducing ends of the CNCs combine to form a star‐shaped supramolecular structure, which decreases in temperature and disintegrates into individual CNCs (Figure 12e). This opens the way for the supramolecular assembly of nanocrystals with controlled geometry for various applications such as reaction materials, pore‐controlled membranes, or the release of target molecules. Cellulose nanocrystals can be applied to Pickering emulsions by enhancing their amphiphilicity through modification. Tang et al.^[^ ^177^ ^]^ introduced CNCs for end‐group modification in hydrophobic chains (polystyrene) to improve their amphiphilicity and increase the stability of the oil‐water interface. Similarly, Du et al.^[^ ^178^ ^]^ modified an 18‐C alkyl long chain at the reducing end. Due to the stability of the pH reaction of the carbon‐nitrogen bond between the 18‐C alkyl chain and CNCs, the introduced 18‐C alkyl chain can be completely decomposed under suitable acid conditions, so that the resulting Pickering emulsion has PH‐triggered deemulsification characteristics.

## 4 Fundamental Fabrication and High‐Value Applications of Janus CNMs

### 4.1 Intrinsic Janus Structure and Feature of CNMs for Absorbent

Cellulose nanomaterials (CNMs), including cellulose nanofibrils (CNFs) and holocellulose nanocrystals (holoCNCs), exhibit unique properties that suggest the presence of a microscopic Janus structure and Janus features, which appear to be intrinsic. Here is some evidence.

**Figure 13:**
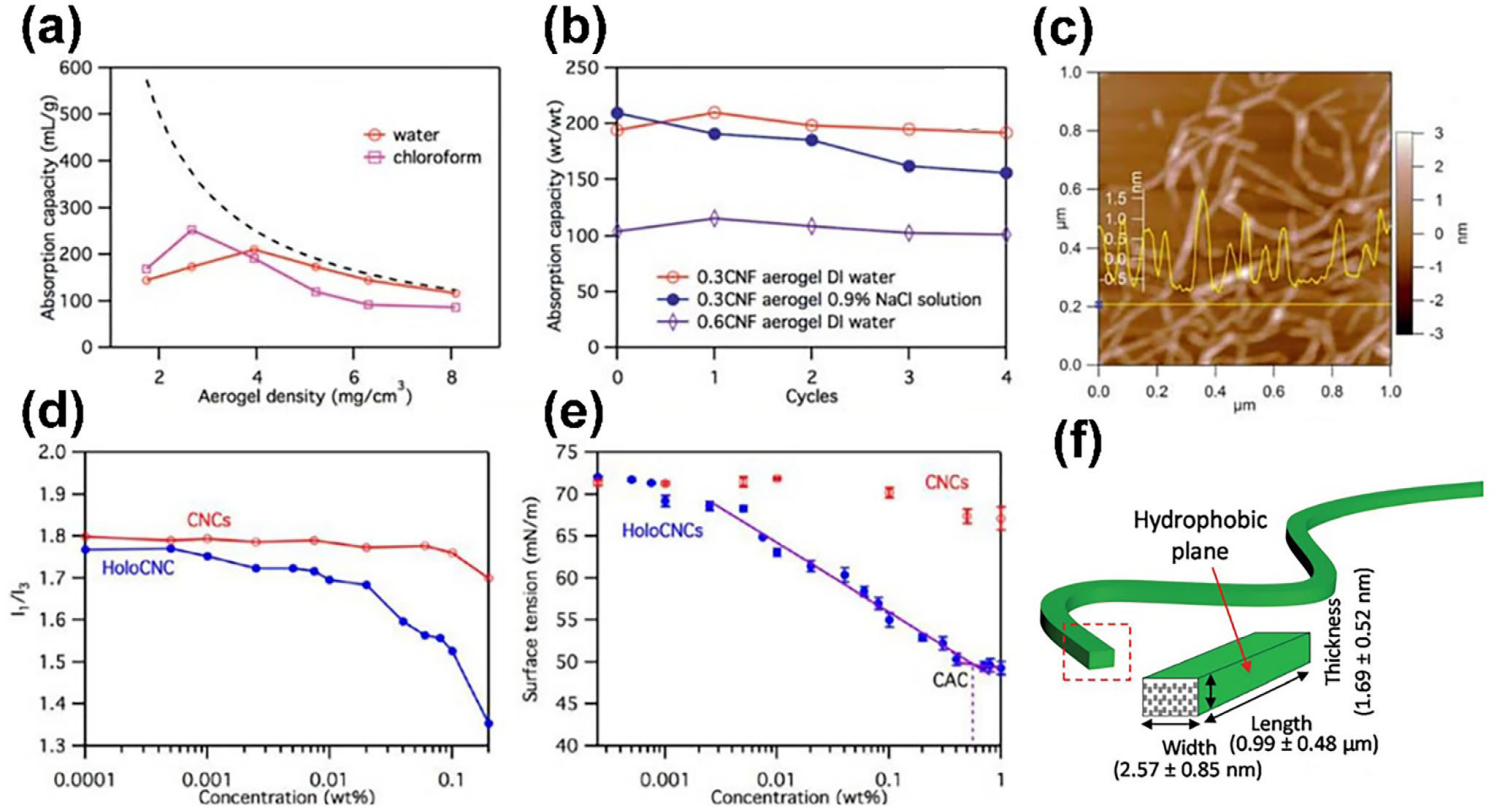
Water saturated CNF aerogels: a) Water and chloroform absorption versus CNF density; dashed line shows calculated values (porosity/density, constant volume). b) Cyclic absorption of water and 0.9% NaCl by 0.3 CNF (4.0 mg cm^−3^) and 0.6 CNF (8.1 mg cm^−3^) aerogels. c) AFM height image and profile of TEMPO‐oxidized, mechanically blended CNFs.^[^ ^191^ ^]^ d) Aqueous CNCs and holoCNCs suspensions: I_1_/I_3_ ratio of pyrene emission spectrum and e) surface tension.^[^ ^192^ ^]^ f) Simplified model of cellulose with (200) hydrophobic sides (C–H) and (110)/(1‐10) hydrophilic surfaces (‐OH); dimensions from AFM/TEM.^[^ ^193^ ^]^

Jiang et al.^[^ ^191^ ^]^ reported that CNFs exhibit intrinsic amphiphilicity, characteristic of a Janus‐like structure. Each anhydroglucose unit in the cellulose chain contains three hydrophilic hydroxyl groups surrounding a relatively hydrophobic pyranose ring, endowing CNFs with affinity for both polar and nonpolar liquids (**Figure** **13**a). For instance, a 0.3 CNF aerogel can absorb up to 210 g g^−1^ of water and 192 mL g^−1^ of nonpolar chloroform. This dual absorption behavior strongly supports the Janus nature of CNFs. As shown in Figure 13b, the absorption capacities of CNF aerogels for water and chloroform clearly demonstrate their interaction with both types of solvents. Furthermore, the AFM image in Figure 13c reveals the fine lateral dimensions of CNFs. Given their amphiphilic nature, it can be inferred that different crystallographic planes on the CNF surface exhibit distinct affinities, further supporting the Janus‐like structural model.

In another study, holocellulose nanocrystals (holoCNCs), derived from traditional sulfuric acid‐hydrolyzed CNCs, also exhibit amphiphilic characteristics.^[^ ^192^ ^]^ The surface properties of holoCNCs are influenced by residual components such as lignosulfonates, hemicellulose, and silica. Steady‐state fluorescence spectroscopy using pyrene as a polarity probe indicates that holoCNCs are less polar than CNCs and can create more hydrophobic microenvironments. For instance, the I_1_/I_3_ value of pyrene in a 0.2% holoCNC suspension reaches 1.35, with increasing values at higher concentrations, suggesting enhanced hydrophobicity. Moreover, the surface tension of aqueous holoCNC suspensions decreases significantly with concentration, reaching a plateau of 49.2 mN/m above the critical aggregation concentration of 0.57%. These findings support the presence of amphiphilic, Janus‐like features in holoCNCs.

Figure 13d illustrates the I₁/I₃ ratios and surface tension profiles of CNC and holoCNC suspensions, providing direct evidence of their amphiphilic nature. Additionally, as shown in Figure 13e, holoCNCs demonstrate superior stabilization of oil‐in‐water (O/W) emulsions compared to CNCs. The larger average droplet sizes in holoCNC‐stabilized emulsions (1.2–1.6 µm) versus CNC‐stabilized ones (0.6–0.8 µm) further suggest that holoCNCs more effectively interact with both oil and water phases, reinforcing their Janus‐like amphiphilic behavior.

TEMPO‐oxidation‐assisted mechanically defibrillated CNFs used for graphene exfoliation, as reported by Xu et al.,^[^ ^193^ ^]^ further support the Janus‐like nature of CNFs. While early studies primarily described their behavior as amphiphilic, current findings reveal clear structural asymmetry. The CNFs possess hydrophilic surfaces with charged carboxylates and hydrophobic (200) planes. During exfoliation, these hydrophobic planes interact with graphite, while the hydrophilic (110)/(1‐10) planes and surface charges facilitate graphene delamination and dispersion.

Figure 13f illustrates the Janus model of CNFs. Surface tension measurements show a non‐monotonic trend with concentration, reflecting complex water interactions characteristic of amphiphilic materials. Moreover, graphene/CNF nanopapers exhibit moisture‐responsive bending: water absorption by hydrophilic domains causes expansion, while the hydrophobic graphene aids recovery upon drying. The observed cyclic deformation highlights the functional significance of CNFs' Janus‐like properties.

### 4.2 Preparation of Janus Cellulose Nanomaterials

**Figure 14:**
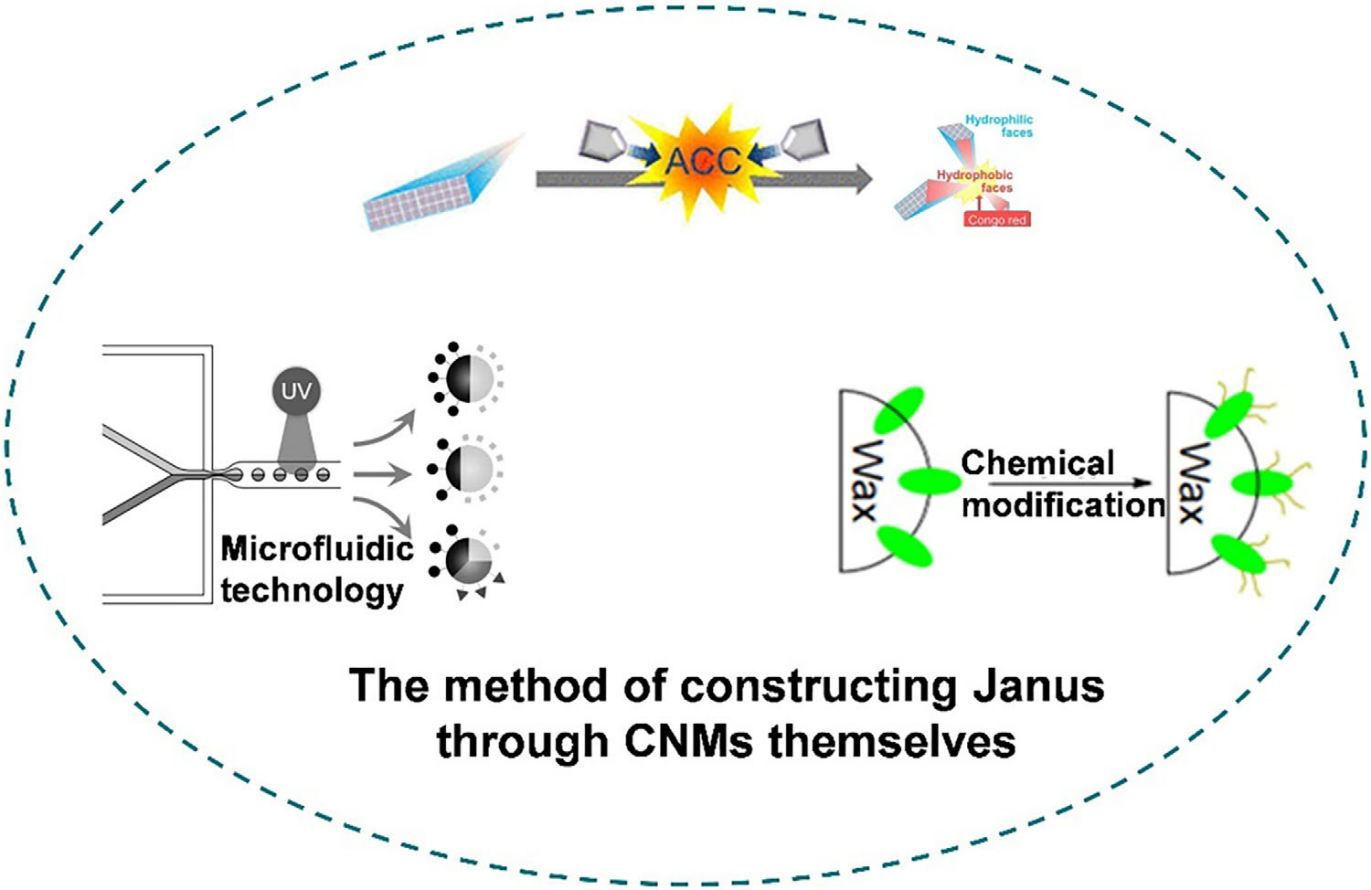
Schematic diagram of the main preparation techniques for Janus CNMs.

Janus cellulose nanomaterials require deliberate structural or chemical asymmetry beyond the inherent amphiphilicity of cellulose to achieve true Janus properties. While the natural amphiphilicity arising from hydrophilic hydroxyl groups and hydrophobic pyranose rings provides a foundation, additional modifications are typically necessary to create distinct interfacial behaviors. For cellulose nanofibrils (CNFs), surface charges introduced through methods like TEMPO‐mediated oxidation can enhance Janus characteristics by increasing hydrophilicity on specific surfaces, as demonstrated in graphene dispersion systems. Holocellulose nanocrystals (holoCNCs) exhibit more complex behavior due to non‐cellulosic components like lignosulfonate that modify regional hydrophobicity. Current preparation methods focus on either exploiting native anisotropy through techniques like aqueous counter collision (which exposes different crystal planes, as illustrated in **Figure** **14** or inducing asymmetry via selective surface modification, directional adsorption, microfluidics, or interfacial assembly. Composite approaches including self‐assembly,^[^ ^50^, ^194^ ^]^ electrospinning,^[^ ^71^ ^]^ directional adsorption,^[^ ^20^ ^]^ freeze‐dry,^[^ ^69^, ^195^ ^]^ microfluidics,^[^ ^42^ ^]^ spray coating,^[^ ^196^ ^]^ selective surface modification,^[^ ^28^, ^38^, ^49^, ^74^ ^]^ and solution casting method^[^ ^51^, ^80^ ^]^ face challenges in achieving precise interfacial control while maintaining material integrity. These Janus‐specific fabrication strategies, distinct from conventional cellulose material preparation, enable tailored surface properties for applications requiring asymmetric interfacial behavior. The development of reliable methods to characterize and control this asymmetry remains crucial for advancing functional Janus cellulose nanomaterials.

For example, solution casting is the most commonly used method for fabricating cellulose‐based composite films due to its simplicity, low cost, and high film quality.^[^ ^51^ ^]^ However, it often yields randomly packed structures with limited long‐range order, where membrane quality is highly dependent on CNC suspension purity. Layer‐by‐layer (LBL) assembly offers precise thickness control but may suffer from interfacial complexity and contamination.^[^ ^91^ ^]^ Spray‐coating enables rapid deposition on porous substrates,^[^ ^196^ ^]^ while spinning techniques allow for anisotropic architectures through shear‐induced fibril alignment.^[^ ^71^ ^]^ Each method presents unique advantages and challenges, requiring careful optimization to achieve films with desirable structural integrity, uniformity, and functional performance.^[^ ^112^ ^]^

The microfluidics method can generate composite droplets with pre‐designed and controllable sizes. The periodic rupture of a liquid line composed of two immiscible liquid flows in parallel produces Janus and three‐phase droplets, with their morphology controlled by the relative flow rate of the corresponding liquids.^[^ ^42^ ^]^ We have previously discussed freeze‐dry technology and chemical modification technology in the theoretical section, but will not elaborate on them here. Simultaneously, we have summarized the uses and methods of Janus CNMs in different forms in **Table** **3**.

**Table 3:** Fabrication methods and applications of Janus cellulose nanoparticles, threads, and membranes.

Morphology	Preparation method	Application
Nanoparticles	Selective surface modification	Flexible sensing^[^ ^74^ ^]^
	Selective surface modification	Pickering interface catalytic and stable^[^ ^28^ ^]^
	Microfluidics	Programmable active soft matter^[^ ^42^ ^]^
Nanorods	Selective surface modification	Flexible sensing^[^ ^74^ ^]^
	The water flushes opposite	Pro‐hydrophobic^[^ ^32^ ^]^
	Selective surface modification	Fluid manipulation and controlled adsorption/desorption^[^ ^38^ ^]^
Membranes	Electrospinning	Emulsion separation^[^ ^71^ ^]^
	Freeze drying	Emulsion separation^[^ ^69^ ^]^
	Freeze drying	Thermal management, flame retardant^[^ ^195^ ^]^
	Growth in situ	Emulsion separation, antibacterial^[^ ^49^ ^]^
	Self‐assembly	Flexible electronic materials^[^ ^50^ ^]^
	Self‐assembly	Response membrane^[^ ^194^ ^]^
	Solution casting method	Response executor^[^ ^80^ ^]^
	Solution casting method	Thermal management^[^ ^51^ ^]^

### 4.3 Thermal Management

**Figure 15:**
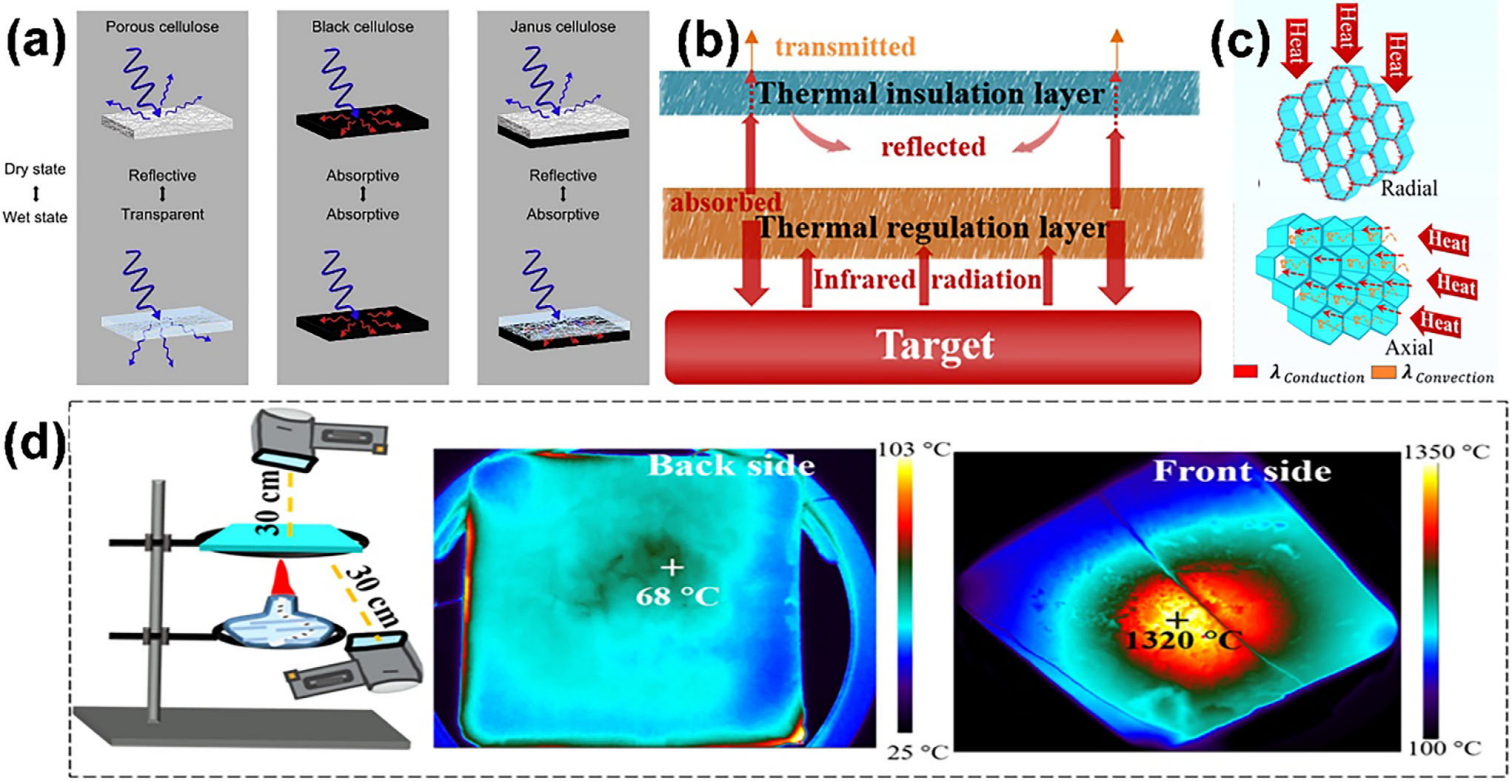
a) Schematic diagram of the absorption and reflection of light radiation by Janus cellulose film in the dry and wet states, with blue and red lines indicating reflection and absorption, respectively.^[^ ^78^ ^]^ b) Schematic diagram of thermal regulation and infrared camouflage mechanism of multifunctional composite materials on targets.^[^ ^77^ ^]^ c) Longitudinal and transverse anisotropic heat conduction mechanism of Janus CNF aerogel.^[^ ^77^ ^]^ d) Thermal insulation and flame‐retardant diagram of Janus CNF aerogel.^[^ ^195^ ^]^ Reprinted with permission from ref.[196] Copyright: 2022, Elsevier; Reprinted with permission from ref.[51] Copyright: 2022, Elsevier; Reprinted with permission from ref.[78] Copyright: 2021, ACS.

Cellulose‐based Janus membranes are mostly thermally managed with asymmetric radiation characteristics on both sides, Yue et al.^[^ ^51^ ^]^ prepared “A‐C‐B” type Janus thermal management membranes using cellulose, MnO_2_, and silver nanowires wrapped by ZnO_2_ nanorods. The cellulose side of ZnO_2_ has high solar radiation reflectivity and high infrared emissivity, which can minimize the heat input of the sun and enhance the heat dissipation effect in the thermal environment. The Ag side has a high solar radiation absorption rate and a low infrared emissivity, which can enhance the heat input of the sun and reduce the loss of heat radiation in cold environments. At the same time, the introduction of porosity into the cellulose structure induces light scattering and makes the material reflective. On the basis of asymmetric radiation on both sides, it is interesting that there is a dynamic relationship between the reversible wetting of liquids such as water in the reflective and transparent state, and when dry, the membrane on the surface of Janus reflects solar radiation in large quantities; When wetted, the underlying membrane reflects a large amount of radiation to dry the upper membrane, which initiates a self‐adapting process that drives the water to evaporate and dry the structure so that it reflects again. The mechanism is shown in **Figure** **15**a and complements the application of cellulose in thermal management.^[^ ^78^ ^]^

Chen et al.^[^ ^77^ ^]^ develop a multifunctional Janus cellulose composite material (CNFphobic/PPy@CNFphilic‐PEG) for on‐demand thermal management by combining CNF aerogel, polypyrrole (PPy), and polyethylene glycol (PEG), which provides electrothermal conversion, thermal regulation, energy storage, and infrared camouflage. The original asymmetric wettability of aerogel ensures the subsequent controlled modification of PPy. Thanks to the excellent electrical conductivity of PPy and the latent heat storage properties of PEG, the multifunctional composites of the PPy@CNFphilic‐PEG layer show excellent electric heating and thermal regulation properties. In personal thermal management, the layer CNF phobic has low thermal conductivity (26.3 mW m^−1^ K^−1^), which can effectively prevent heat loss in the internal heating layer and provide joule heat for heating according to demand under low applied voltage. The composite material CNFphobic/PPy@CNFphilic‐PEG is based on the synergistic mechanism of infrared radiation absorption and heat insulation: the PPy@CNFphilic‐PEG layer phobic serves as the thermal regulation layer to absorb infrared radiation. And the outer layer of CNFphobic serves as the heat insulation layer to prevent most heat transfer contributed by its porous structure and low thermal conductivity (Figure 15b)

Yan et al.^[^ ^195^ ^]^ prepared a “strong and soft” Janus NFC aerogel by one‐way freeze‐drying method. NFC‐Si‐T aerogel exhibits high axial Young's modulus and high flexibility and can rebound quickly in liquid nitrogen (−196 °C). The radial center temperature of the sample is 17.41 °C lower than the axial center temperature. Because the heat convection and heat conduction of the solid phase in the axial direction are greatly increased than in the radial direction (Figure 15d). In addition, NFC‐Si‐T aerogel has anisotropic thermal insulation performance with low average thermal conductivity (0.028–0.049 W m^−1^ K^−1^) due to its structure, as shown in Figure 15c. After the introduction of MTMS and TsOH, NFC‐Si‐T can better block the heat and volatile pyrolysis products of cellulose.

### 4.4 Pressure Sensing

Additionally, cellulose exhibits unique electrical and electronic properties. CNMs, known for their high dielectric properties (ε > 6) and strong response to electric fields, are commonly combined with polymers like polyethylene oxide (PEO) to form uniformly dispersed colloidal systems.^[^ ^197^ ^]^ These materials can sense voltage, rotate, and self‐assemble. In an electric field of ≈15 kV, CNCs and CNFs align in parallel.^[^ ^171^ ^]^ Xu et al.^[^ ^198^ ^]^ observed a crystal structure resembling grilled meat skewers in PEO/CNC and PEO/CNF nanofibers. CNMs exhibit proton conductivity, enabling them to transport protons while blocking electrons. Research by Buyer et al.^[^ ^199^ ^]^ indicates that CNCs possess higher conductivity (due to more charge carriers) than CNFs. At 100% relative humidity, the maximum conductivity of CNF paper film at 100 °C is 0.05 mS cm^−1^, whereas CNC paper film shows a conductivity of 4.6 mS/cm at 120 °C. Bras et al.^[^ ^200^ ^]^ investigated the electrical properties of CNMs and found that solid‐state characteristics such as crystallinity and water absorption significantly influence conductivity, which increases notably with higher humidity and frequency.

**Figure 16:**
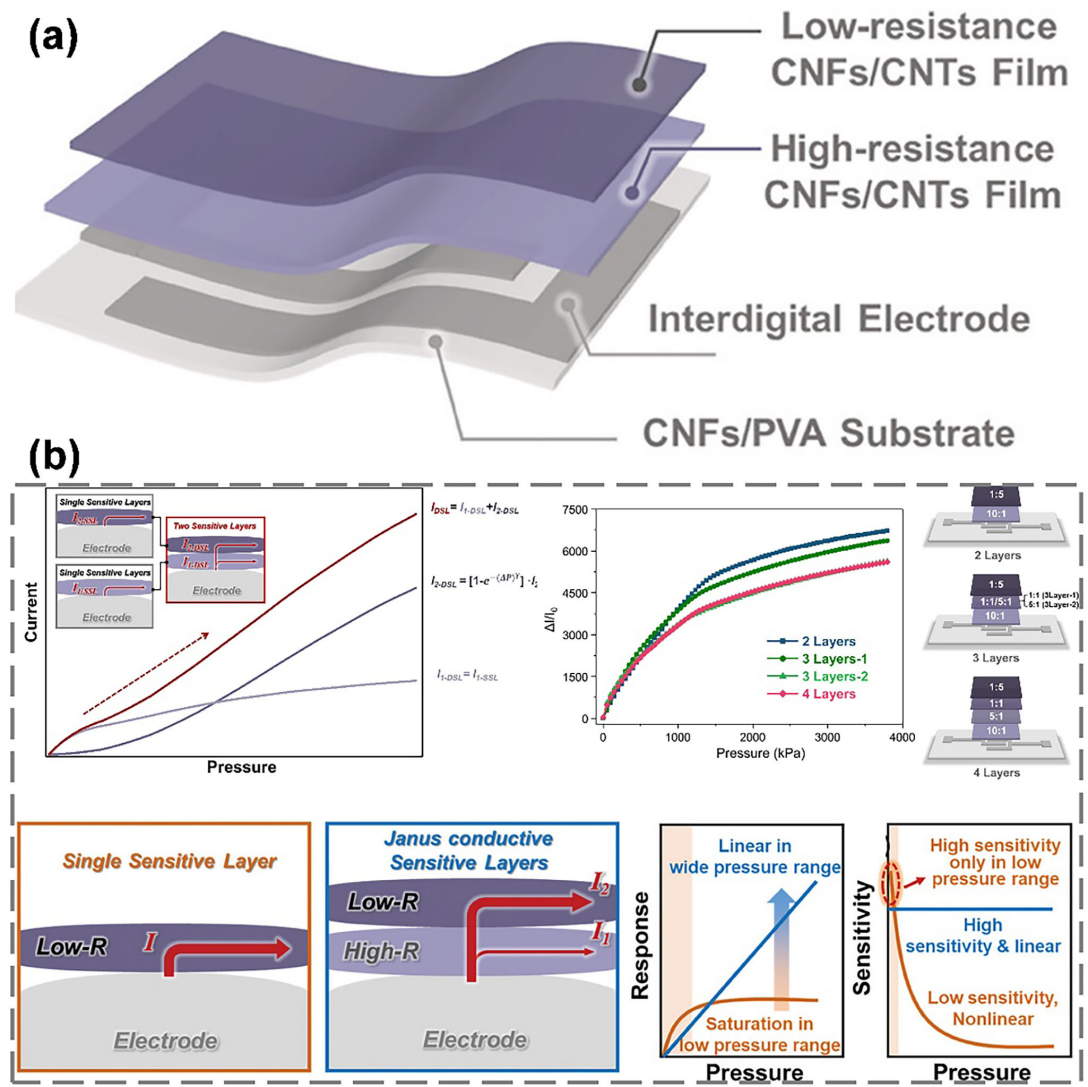
a) Schematic diagram of the pressure sensor structure. b) Comparison of pressure and current response behavior between the Janus dual‐sensing layer and single‐layer and multi‐layer structures.^[^ ^201^ ^]^ Reprinted with permission from ref.[199] Copyright: 2024, Wiley.

Lin et al.^[^ ^201^ ^]^ developed a paper‐based pressure sensor using CNF paper as the substrate and a CNF/carbon nanotube (CNT) composite as the sensitive layer. They fabricated CNF/polyvinyl alcohol (PVA) and CNF/CNT paper through a simple solution casting method. The addition of PVA to the CNF matrix enhanced the mechanical strength and toughness of the paper, allowing it to withstand higher mechanical stress. In the single‐sensitive layer structure, as external pressure increases, the contact interface between the electrode and CNF/CNT paper expands. However, the change in interface contact resistance is minimal, causing the resistance to saturate quickly at lower pressures. Therefore, the sensor exhibits high sensitivity only in the low‐pressure range, with poor linearity at higher pressures (**Figure** **16**a).

To verify the advantages of the Janus configuration, researchers found that increasing the conductivity difference between the dual‐sensitive layers enhances the sensor's sensitivity (where sensitivity *S* is defined as the ratio of current response *∆I/I_0_* to the applied pressure change *∆P*). They further demonstrated that a multi‐layer configuration of the sensitive layers reduces the rate of conductivity gradient change and increases the interface contact resistance, which in turn limits the improvement of sensor sensitivity (as shown in Figure 16b, illustrating the relationship between pressure and sensitivity). In contrast, the dual‐sensitive layer structure features two contact interfaces, where the current transmission path switches between the two sensitive layers as pressure changes. This allows the sensor to exhibit a broader range of resistance variations and a slower resistance response, leading to a wider pressure sensing range and improved linearity (The pressure response curve in Figure 16b).

### 4.5 Electromagnetic Shielding

Electromagnetic interference (EMI) shielding materials are essential for reducing electromagnetic radiation pollution.^[^ ^202^ ^]^ While traditional metal shielding materials offer excellent thermal and electrical conductivity, they suffer from challenges such as difficult processing, poor flexibility, high density, and susceptibility to corrosion.^[^ ^203^ ^]^ Conductive polymer composites, on the other hand, typically require high filler loads to achieve optimal thermal conductivity and EMI shielding, which compromises their mechanical properties and flexibility, thus limiting their use in flexible wearable devices.^[^ ^204^ ^]^ CNMs are widely used for multifunctional EMI shielding due to their environmental friendliness, low cost, high mechanical strength, and potential for thermal conductivity. For instance, pure CNF exhibits a thermal conductivity of 1.31 W·m⁻¹·K⁻¹.^[^ ^205^ ^]^

**Figure 17:**
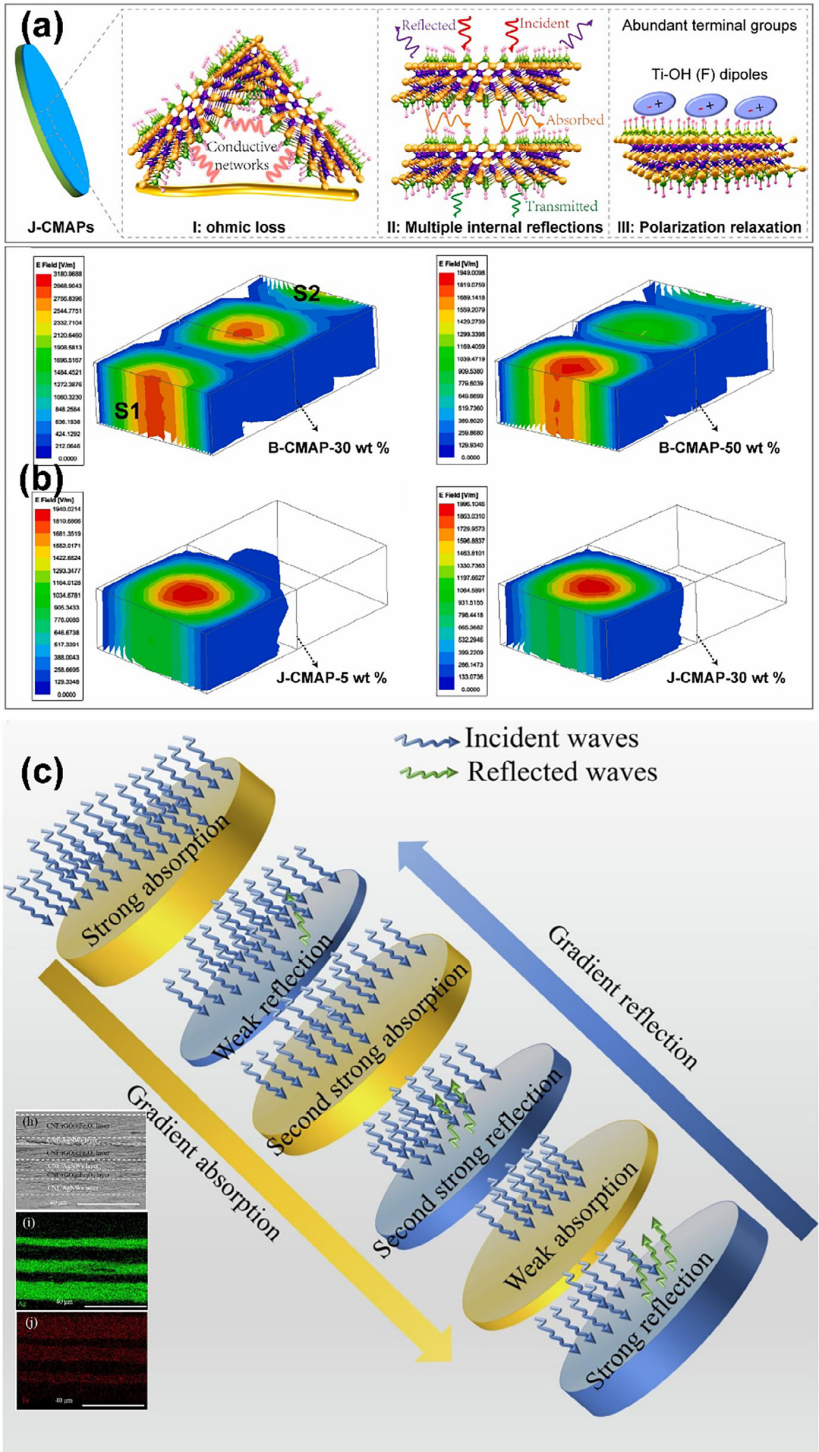
a) Schematic illustration of the EMI shielding mechanism of the J‐CMAPs. b) Simulation of J‐CMAPs and B‐CMAPs with various MXene/AgNWs contents shielding EM waves enabled by HFSS simulation in X‐band, respectively.^[^ ^34^ ^]^ c) Electromagnetic shielding mechanism of multi‐layer Janus nanomaterials.^[^ ^207^ ^]^ Reprinted with permission from ref.[204] Copyright: 2023, Elsevier; Reprinted with permission from ref.[206] Copyright: 2022, Elsevier.

The electromagnetic wave shielding mechanism of J‐CMAPs involves multiple processes, including reflection, energy dissipation in conductive networks, and internal reflection. Initially, electromagnetic waves are partially reflected due to surface impedance mismatch. They then interact with the high‐density electron carriers in the MXene/AgNWs network, resulting in significant Ohmic losses and energy attenuation. Internal reflection between MXene nanosheets further promotes wave energy attenuation until complete absorption. Surface defects and end groups generate local dipoles, enhancing shielding performance (**Figure** **17**a).^[^ ^34^, ^206^ ^]^ In contrast, Blended‐CMAP (B‐CMAP) shows limited shielding effectiveness due to their lower conductivity.

Figure 17b, through HFSS simulation, compares the electric and magnetic field distributions of J‐CMAPs and B‐CMAPs to explain their EMI shielding mechanism. In B‐CMAPs with 30 wt.% MXene/AgNWs, EM waves easily pass through with weak shielding ability. With 50 wt.% MXene/AgNWs, the electric and magnetic field strength is reduced, but the shielding effectiveness remains limited. Conversely, J‐CMAPs exhibit a strong electric field response and significant EMI shielding at just 5 wt.% MXene/AgNWs content. As the MXene/AgNWs content increases, the distribution of electric and magnetic fields diminishes, confirming that conductivity loss is the dominant factor in EM wave attenuation.

Hu et al.^[^ ^207^ ^]^ improved the electromagnetic interference (EMI) shielding and mechanical properties of cellulose composite films by using an asymmetric gradient alternating multilayer structure. The 6‐layer nanocomposite film achieved an impressive EMI shielding effectiveness of up to 112.9 dB, with an average EMI SE of 104 dB in the X‐band. This exceptional performance is attributed to its high conductivity and novel shielding mechanism, where electromagnetic waves undergo both gradient absorption and gradient reflection across the film layers (Figure 17c). Interestingly, although the conductivity of the asymmetric gradient alternating multilayer films decreases with increasing layers, the EMI shielding performance improves, highlighting the complex relationship between conductivity and shielding efficacy in these structures.

### 4.6 Optoelectronic Devices

In addition to the optical properties of cellulose itself, our previous research revealed that sulfonated cellulose nanocrystals can be mixed with perovskite precursor solutions, leading to in situ perovskite growth in CNC. Vacuum filtration and drying resulted in a perovskite paper with excellent luminescence performance.^[^ ^208^ ^]^ Owing to the presence of abundant hydroxyl (─OH) and sulfate (─OSO_3_Na) groups, CNCs are expected to exhibit a high electronegativity to complex with cations, that is, CH_3_NH_3_^+^, Pb^2+^ in metallic halide perovskites. Cellulose replaces traditional organic ligands such as oleic acid and oleylamine to stabilize perovskite while passivating defects. Improved the luminescence stability and UV resistance of perovskite. For example, the relative photoluminescence intensity of PQDP remains over ≈90% under continuous ultraviolet (UV, 16 W) irradiation for 2 months.^[^ ^209^ ^]^ Li et al.^[^ ^210^ ^]^ prepared panchromatic perovskite paper by manipulating the types and proportions of perovskite halogen atoms mixed with cellulose. Recently, Kim et al.^[^ ^211^ ^]^ directly sprayed perovskite precursor onto cellulose paper to form a layer of perovskite, preparing a large area of Janus‐like perovskite thin film. By controlling the volume of the precursor, the brightness can be accurately adjusted.

Cellulose nanomaterials have the potential to be used as substrates, color conversion layers, and even luminescent layers in light‐emitting diodes (LEDs).^[^ ^2^, ^208^ ^]^ However, the high thermal expansion resulting from the heat generated by devices may deform the device if plastic is used as the substrate. Therefore, cellulose nanomaterials‐based plastic nanocomposites appear to offer good thermal stability and high light transmittance. Cellulose can be doped with conductive polymer fibers to create transparent conductive materials, serving as an alternative to Indium‐Tin Oxide in LED.^[^ ^212^ ^]^ By doping silver nanowires, films with optoelectronic properties and resistance as low as 2.4 ohm square^−1^ can be obtained. Previous work has introduced the concept of Janus into the design of semiconductors, and in addressing the issue of carrier mobility, Janus structures do exhibit extraordinary charge transport characteristics.^[^ ^213^ ^]^ This also provides a possible optoelectronic device structure for cellulose.

## 5 Conclusion

In conclusion, our study has extensively investigated the Janus properties of cellulose nanomaterials, with a particular focus on the potential to directly derive Janus materials from cellulose molecular chains. By summarizing various molecular‐level driving forces and regulatory strategies—including hydrogen bonding, hydrophobic interactions, and the Aqueous Counter Collision mechanism—we have elucidated the fundamental mechanisms underlying the Janus characteristics of cellulose nanomaterials. Through the integration of molecular simulation data from both crystalline and amorphous regions, we have further demonstrated the structural and functional implications of these properties. Moreover, we emphasize the role of chemical modifications to cellulose side chains and reducing ends, which not only significantly enhance their Janus properties but also provide valuable strategies for advancing cellulose nanomaterials in high‐value applications. These modifications enable the fine‐tuning of the hydrophilic and hydrophobic balance, thus allowing for the tailoring of cellulose nanomaterials for specific applications, such as in pressure sensing, electromagnetic shielding, and energy management systems. Our research suggests that cellulose nanomaterials can serve as a promising platform for the environmentally friendly fabrication of Janus materials, using straightforward methodologies that are both cost‐effective and scalable. By further optimizing these approaches, cellulose nanomaterials have the potential to play a key role in the development of sustainable, high‐performance Janus materials for a range of industrial and technological applications.

Over the past few decades, nanotechnology and synthetic chemistry have successfully engineered nanoscale building blocks, such as functional molecules or colloidal nanostructures, on demand. Inspired by nature, these building blocks can be precisely assembled to create layered mesoscopic structural materials with novel and advanced functions. The potential applications of this technology range from energy conversion to storage, catalysis, and smart surfaces. However, current methods for surface modification of cellulosic materials face challenges in terms of control and scalability, making it difficult to implement highly customized Janus structures on these materials. We have summarized the following challenges:

1. Streamlining Preparation Methods: The creation of Janus cellulose materials with precise asymmetric structures currently requires complex and multi‐step processes. Future research should focus on developing more efficient and scalable fabrication techniques that can precisely regulate the properties of different regions within the material. Achieving fine control over the structural asymmetry will be crucial for expanding the applicability of Janus cellulose materials in high‐performance devices.
2. Enhancing Interface Compatibility: Integrating functional components from different regions of Janus cellulose requires ensuring compatibility at the interface. Further investigation is needed into the molecular interactions at these interfaces, with a focus on minimizing adverse effects such as phase separation or inefficient interfacial bonding. Techniques to improve interfacial compatibility will allow the seamless integration of multiple functionalities, facilitating the development of multifunctional Janus cellulose materials.
3. Controlling Self‐Assembly Processes: The self‐assembly process is key to creating Janus materials from cellulose nanomaterials. However, controlling the self‐assembly behavior remains a significant challenge. Future studies should intensively explore the role of intermolecular interactions—such as hydrogen bonding, hydrophobic forces, and van der Waals interactions—in driving self‐assembly. Understanding how to manipulate these forces will enable precise control over the assembly process, leading to more consistent and predictable formation of Janus structures.

## Conflict of Interest

The authors declare no conflict of interest.
